# Highly accessible AU-rich regions in 3’ untranslated regions are hotspots for binding of regulatory factors

**DOI:** 10.1371/journal.pcbi.1005460

**Published:** 2017-04-14

**Authors:** Mireya Plass, Simon H. Rasmussen, Anders Krogh

**Affiliations:** Section for Computational and RNA Biology, Department of Biology, University of Copenhagen, Ole Maaløes Vej 5, Copenhagen, Denmark; Center for Genomic Regulation, SPAIN

## Abstract

Post-transcriptional regulation is regarded as one of the major processes involved in the regulation of gene expression. It is mainly performed by RNA binding proteins and microRNAs, which target RNAs and typically affect their stability. Recent efforts from the scientific community have aimed at understanding post-transcriptional regulation at a global scale by using high-throughput sequencing techniques such as cross-linking and immunoprecipitation (CLIP), which facilitates identification of binding sites of these regulatory factors. However, the diversity in the experimental procedures and bioinformatics analyses has hindered the integration of multiple datasets and thus limited the development of an integrated view of post-transcriptional regulation. In this work, we have performed a comprehensive analysis of 107 CLIP datasets from 49 different RBPs in HEK293 cells to shed light on the complex interactions that govern post-transcriptional regulation. By developing a more stringent CLIP analysis pipeline we have discovered the existence of conserved regulatory AU-rich regions in the 3’UTRs where miRNAs and RBPs that regulate several processes such as polyadenylation or mRNA stability bind. Analogous to promoters, many factors have binding sites overlapping or in close proximity in these hotspots and hence the regulation of the mRNA may depend on their relative concentrations. This hypothesis is supported by RBP knockdown experiments that alter the relative concentration of RBPs in the cell. Upon AGO2 knockdown (KD), transcripts containing “free” target sites show increased expression levels compared to those containing target sites in hotspots, which suggests that target sites within hotspots are less available for miRNAs to bind. Interestingly, these hotspots appear enriched in genes with regulatory functions such as DNA binding and RNA binding. Taken together, our results suggest that hotspots are functional regulatory elements that define an extra layer of regulation of post-transcriptional regulatory networks.

## Author summary

All the cells in a given organism contain the same genome, yet their phenotype can be very diverse. The vast majority of this diversity arises from the differences in the expression of genes and proteins in them. One of the main mechanisms involved in controlling the protein and mRNA repertoire in cells is post-transcriptional regulation. The recent development of high-throughput sequencing techniques gives us now an unprecedented opportunity to investigate how post-transcriptional regulation works and which are the elements involved in defining the final set of mRNAs and proteins inside cells. In this work, we have performed a comprehensive computational analysis of several post-transcriptional regulators in a commonly used human cell line in order to understand which factors are involved in post-transcriptional regulation and how they coordinate their function. The results of our analysis show that this process is orchestrated around small regions in the mRNAs where many regulators bind and may compete with each other to regulate the mRNAs. The investigation and characterization of these regions gives us insight into the underlying combinatorial control that causes gene expression to differ across cell types and in diseases.

## Introduction

Post-transcriptional regulation is the set of mechanisms and processes that control gene expression at the RNA level and affect the properties and the amount of RNA transcribed and translated into proteins. This regulation is performed mainly by RNA binding proteins (RBPs) and miRNAs, which primarily target the 3’ untranslated region (3’UTR) of transcripts. In animals, miRNAs usually function by promoting translational inhibition and decay of mRNAs [1]. In contrast, RBPs have a wider range of functions and are often involved in multiple post-transcriptional processes.

Although some miRNAs are predicted to target thousands of mRNAs [2], not all of their predicted targets are down-regulated upon miRNA transfection [3], and many seem to be regulated only in certain cellular contexts or under stress conditions [4]. In some cases, miRNAs have even been found to promote translational activation [5] or increase mRNA levels. All these different complex functions suggest that miRNAs and RBPs take part in combinatorial regulation, where the *combination* of factors that bind to an RNA determines its fate.

In humans, more than 1000 RBPs [6,7] and ~2500 miRNAs [8] are involved in this complex regulation. MiRNAs are known to act cooperatively to down-regulate mRNAs when bound close in space [3,9,10]. RBPs can compete for binding to AU-rich elements (AREs) [11] or cooperate in mRNA regulation [12,13]. Moreover, they can compete and collaborate with miRNAs, or even perform opposite functions in different contexts [14]. For instance, AUF1 has been found to both compete with AGO2 for binding to the mRNA and cooperate with it [15]. Similarly, HuR has been found to compete with miRNAs for binding [16,17] but also to cooperate with miRNAs both to stabilize and degrade target mRNAs [18,19].

Previous studies that aimed to decipher the interactions between miRNAs and RBPs have been focusing either on single genes [20–24] or on the interactions between a single RBP and miRNAs using cross-linking immunoprecipitation coupled to high-throughput sequencing (CLIP-seq) data [13,16,17], and only recently these interactions have been explored at a transcriptome-wide scale [25,26]. However, the differences across CLIP protocols [27] and existing computational tools used to analyze the resulting data [28,29] complicates their integration and comparison, which is required for obtaining a picture of their interactions on a global scale. Additionally, CLIP methods typically include a significant amount of noise that can lead to the identification of artifactual binding sites if stringent filtering criteria are not used [30,31].

In this work, we reanalyze a comprehensive collection of CLIP experiments from HEK293 cells to shed light on the complex interactions between RBPs and miRNAs. By using a stringent processing pipeline and integrating multiple datasets, we show that post-transcriptional regulators bind preferentially in specific regions of 3’UTRs, i.e. hotspots, where we find enrichment of both RBP and miRNA target sites. These hotspots, rather than being experimental artifacts as previously thought [26,30], share characteristics typical of other regulatory regions: high sequence conservation, accessibility, and enrichment in AU-rich elements (AREs). Additionally, our results suggest that they might function by favoring competition among regulators. Upon AGO2 knockdown (KD), we observe that the changes in expression level of transcripts that harbor miRNA target sites within hotspots are significantly different from those of transcripts that contain miRNA target sites outside them, which suggests that RBPs binding in hotspots prevents RISC association. Similar changes are observed when an RBP is knocked down, highlighting that competition for binding may occur not only between miRNAs and RBPs but also among RBPs. Interestingly, these hotspots are enriched in genes with roles in transcriptional and post-transcriptional regulation.

Taken together, these results suggest that post-transcriptional regulation is focused in hotspots within 3’UTRs where several regulators can bind in close proximity and modulate the functions of the regulatory network both at transcriptional and post-transcriptional level.

## Results

### RBP binding sites colocalize within 3’UTRs

**Fig 1:**
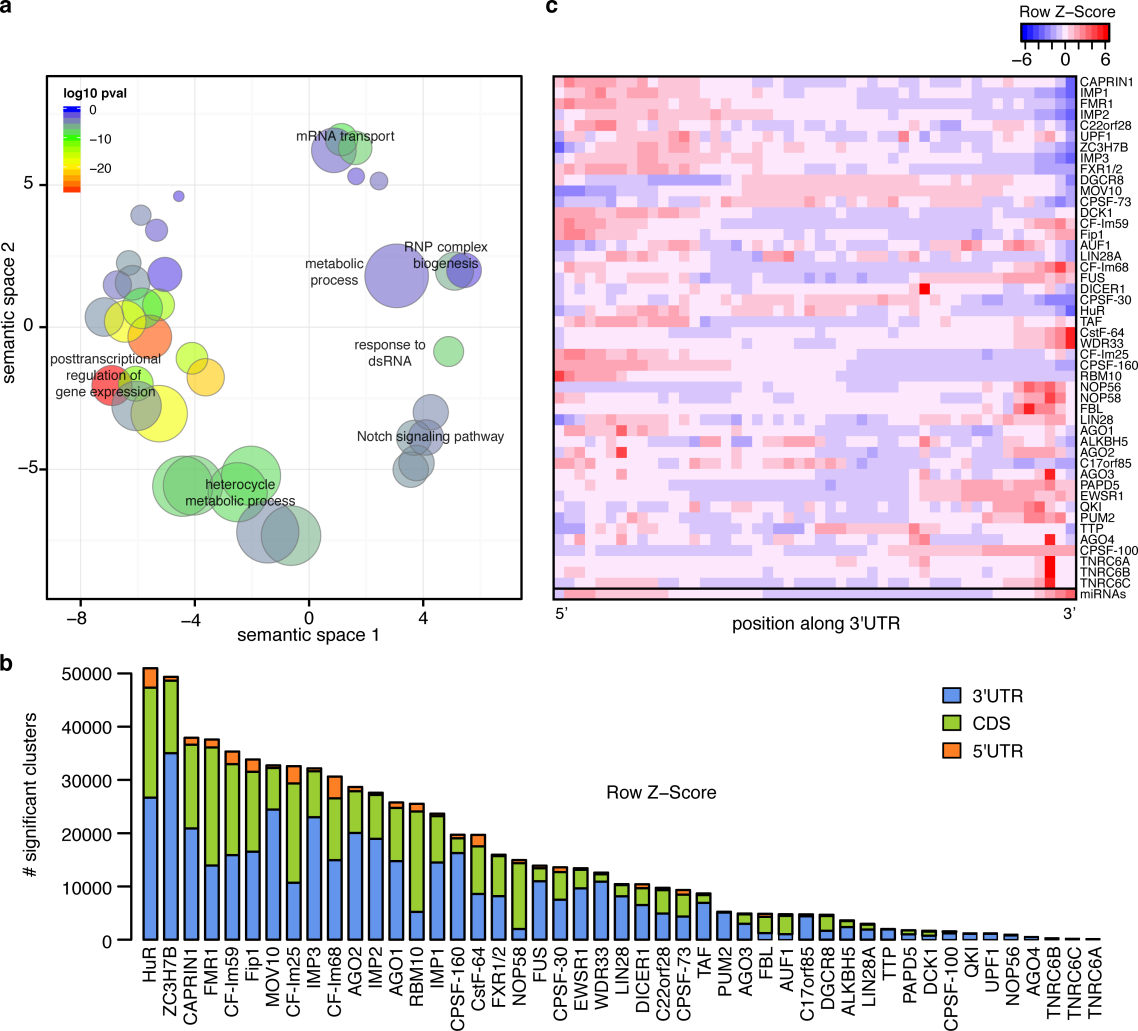
RBPs analyzed are involved in post-transcriptional regulation and bind mainly at 3’UTR edges. **a)** Scatter plot summarizing the most significant GO-terms associated with the RBPs analyzed. Bubble sizes indicate the frequency of the GO-term in the GO database. The color of the bubbles represents how significant the term is in the set of RBPs analyzed. **b)** Distribution of significant clusters (FDR < 0.01) across gene regions. The height of the bars represents the amount of significant clusters in 3’UTRs (blue), coding region (green) and 5’UTR (orange). **c)** Heatmap showing the distribution of significant clusters across standardized 3’UTRs. The colors range from red (higher frequency) to blue (lower frequency).

To investigate the complex interactions between RBPs and miRNAs on a transcriptome-wide scale, we reanalyzed previously published CLIP data for 49 RBPs in HEK293 cells, which correspond to a total of 107 experiments. The list of all the RBPs analyzed along with a brief description from STRING database [32] can be found in S1 Table. An analysis of the functions of these proteins using GO terms revealed that many are involved in similar processes, especially in post-transcriptional regulation of gene expression (Fig 1a; S2 Table).

**Fig 2:**
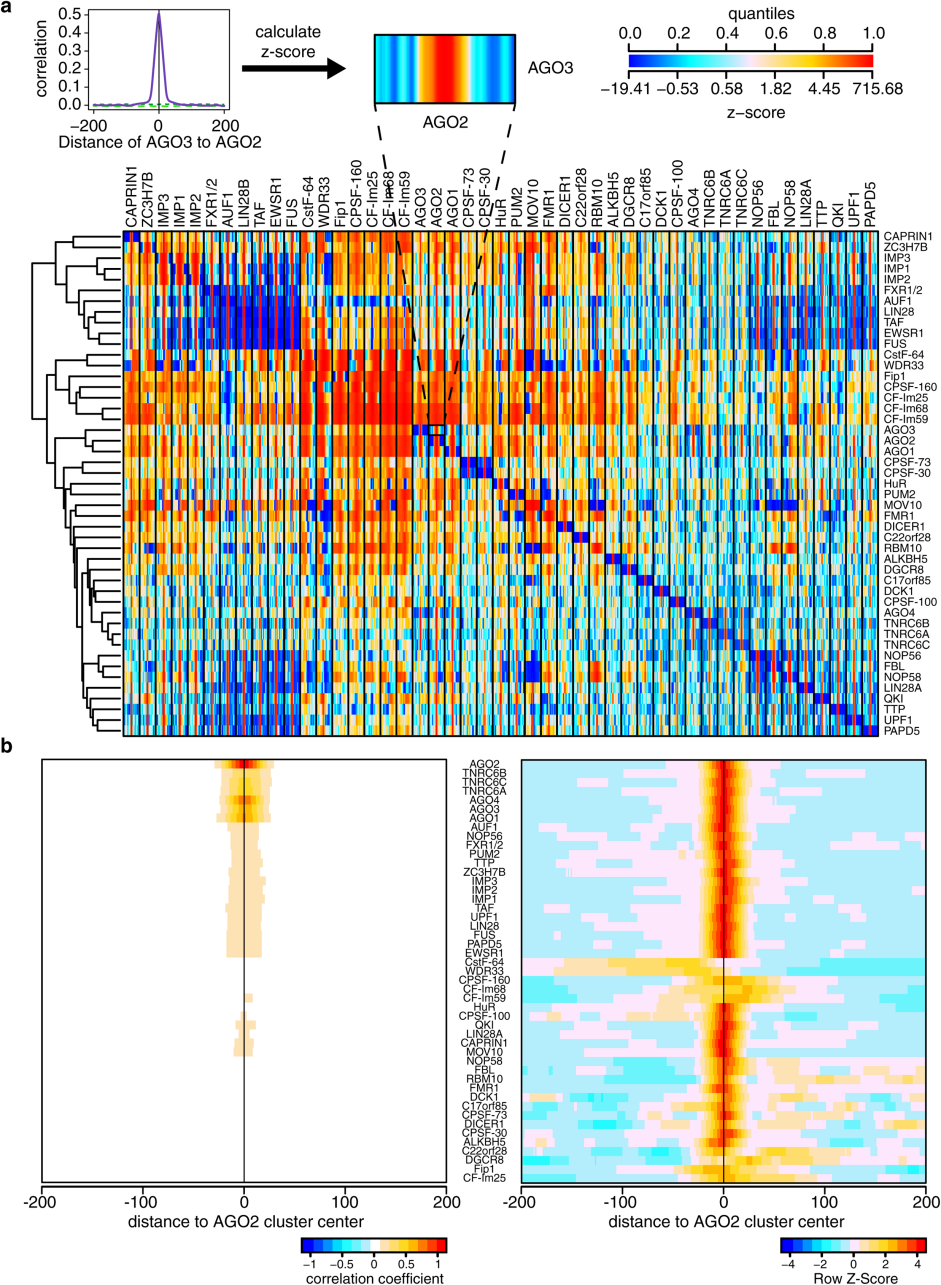
RBP enrichment on 3’UTRs is significantly correlated for most RBP pairs. **a)** Schematic representation of the z-score calculation for each RBP pair (top). Pearson correlation coefficients of cluster enrichments are calculated for each pair of proteins (purple line) in a distance from -200 to 200 nt and compared against the positional correlations obtained from shuffled clusters in the same region (dashed green lines show the top and bottom 5% random distributions). The random clusters are then used to calculate the z-score of the Pearson correlation coefficient at each position. The colors in the main panel represent the z-scores of the correlations and range from blue (lower z-score) to red (higher z-score). All against all positional correlations calculated in this way are summarized in the heatmap (bottom). For each RBP in a column we show the significance of the positional binding of each of the RBPs in the rows around its binding sites. **b)** Zoom on the positional correlations calculated around AGO2 binding sites. The Pearson correlation coefficients of cluster enrichments (left) and their z-scores (right) are shown. The strongest positional correlations are observed around AGO2 binding sites. On the right panel, the z-scores of the correlations are shown, row-normalized.

In order to obtain a set of significant RBP binding sites that can be compared across experiments, all the datasets were reanalyzed using the same pipeline. This pipeline, which has been used previously to identify binding sites from PAR-CLIP and iCLIP datasets [33,34], is focused on improving read preprocessing using custom scripts and mapping using BWA-PSSM [35], which allows to model the high mutation rate observed in CLIP data [36,37], improving both sensitivity and specificity. Moreover, duplicate removal, which usually is performed to discard reads that map to the same location, was done on the sequence of the reads after low quality and adapter removal at the 3’end. Given the high mutation and indel rate observed in CLIP and PAR-CLIP data [36,37], this approach was used in order to keep as many reads that represent RBP binding sites as possible. As expected, a high percentage of the reads confidently mapped from the CLIP datasets contained one or more mutations, especially T to C conversions in PAR-CLIP datasets, which are indicative of crosslinking events (S1 Fig). After mapping, significant clusters were identified using Pyicos [38] (see Materials and Methods and S1 Appendix). For many of the RBPs analyzed, most of the clusters were located on 3’UTRs (Fig 1b), which is consistent with the observation that many of these RBPs are involved in post-transcriptional regulation (S2 Table). On average, each mRNA expressed in HEK293 is bound by 14 different RBPs in its 3’UTR, and 20% of them are bound by more than half of the RBPs analyzed. A detailed analysis of the distribution of RBP binding sites along 3’UTRs showed that most of them bind preferentially towards the 3’UTR edges, where there is also a higher density of miRNA target sites (Fig 1c).To understand if the observed positional bias indicates that the proteins bind in the same regions and therefore interact or compete with each other, we calculated spatial correlations between their cluster enrichments, calculated as the log2 ratio of CLIP over RNA-seq reads (see Materials and Methods). For each pair of proteins, we calculated the Pearson correlation between CLIP enrichment values for each distance between -200 and 200 nt in each 3’UTR. The correlations calculated for these values were averaged over all 3’UTRs, giving us an average spatial correlation profile for the two RBPs (S2 Fig). These correlations were then compared to those obtained from shuffling the clusters to calculate their z-scores at each position (Fig 2a). In each row of Fig 2a, the z-scores of these spatial correlations are shown for each pair of RBPs. The higher the z-score, the more significant the correlation between two RBPs in a particular position is. In 746 out of 1084 pairwise combinations of RBPs (excluding pairing of an RBP with itself), we observed that the highest positional correlation z-score was in the +/- 9 nt interval. This result reflects that for 69% of all RBP pairs, the most significant positional correlation was observed when the clusters of the two RBPs overlap in the same 3’UTR (Fig 2a). If we consider only the positional correlations around AGO2 binding sites, this percentage increases to 89%. As seen in Fig 2b, the strongest positional correlations around AGO2 binding sites were found for other AGO proteins and TNRC6 proteins, which are also part of the RISC complex. Other RBPs display weaker positional correlations with AGO2 although they are highly significant compared to the random expectation.

### RBPs preferentially bind on miRNA target sites

**Fig 3:**
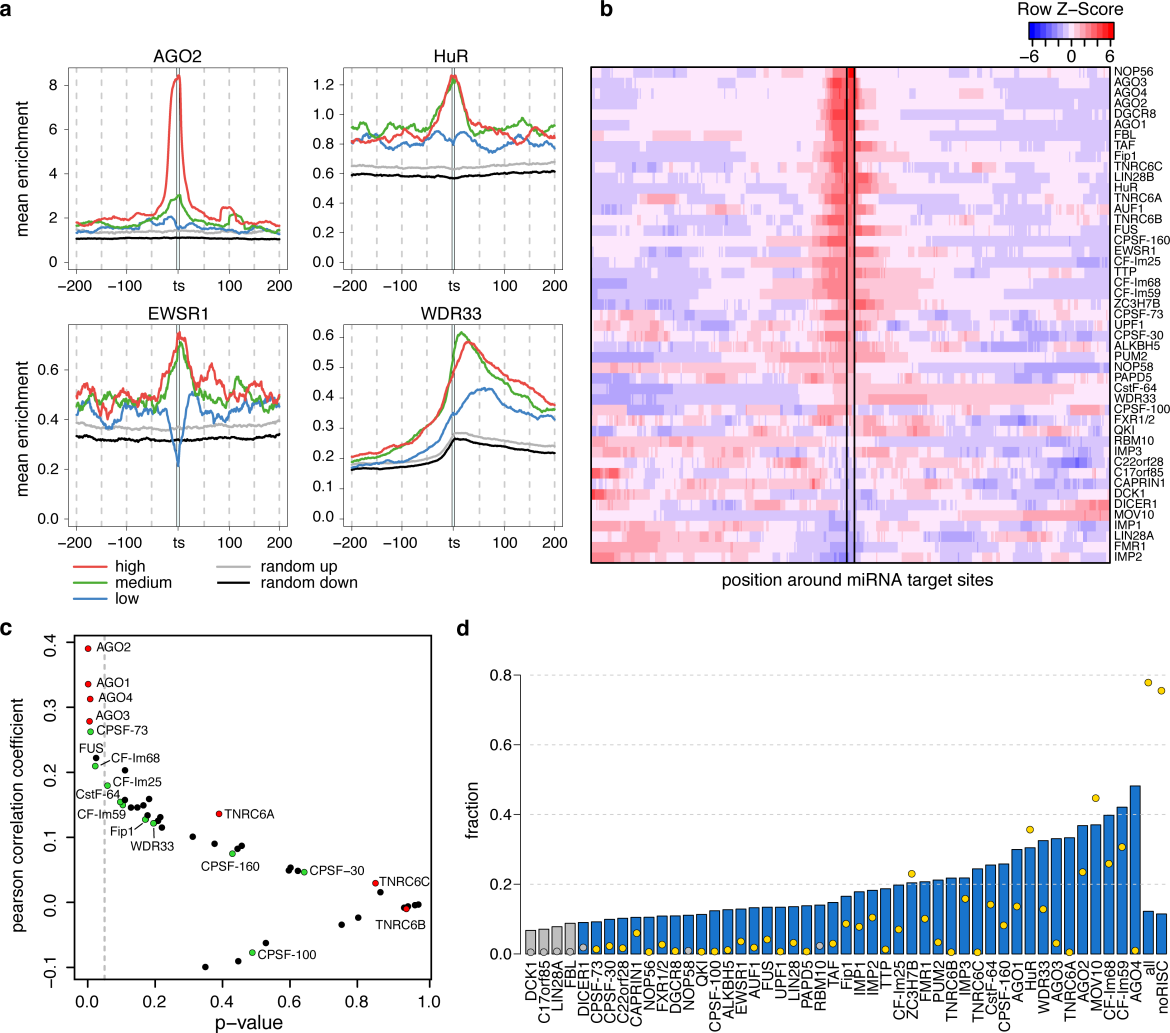
RBP binding sites are enriched on target sites of highly expressed miRNAs. **a)** Characteristic CLIP enrichment profiles for AGO2, HuR, EWSR1 and WDR33 RBPs around miRNA target sites that are highly (red), moderately (green) and lowly (blue) expressed. The grey and the black lines show the maximum and the minimum enrichment values for the 90% confidence intervals around random miRNA target sites. **b)** Heatmap showing the distribution of CLIP enrichment values around *hisites*. The colors range from red (high enrichment) to blue (low enrichment). **c)** Scatter plot summarizing the correlation values between RBP enrichment on miRNA target sites and miRNA expression (y-axis) and their p-values (x-axis). **d)** Barplot summarizing the fraction of RBP clusters on *hisites* (bars) and the fraction of *hisites* overlapped by RBPs (points). Colored bars (blue) and points (yellow) highlight the cases in which the fraction of RBPs or miRNA target sites is higher than expected by chance (empirical p-value < 0.01 using 100 random miRNA target site sets).

The previous analyses demonstrated that RBPs exhibit distinctive binding preferences around AGO2 binding sites. To further evaluate the correlations between RBPs and AGO2, we analyzed the enrichment distribution of their binding sites around predicted target sites of miRNAs expressed in HEK293 cells. 75% of all the target sites from expressed miRNAs in HEK293 were bound by one or more RBPs (S3 Table). As expected, the binding of AGO2 and the other AGO proteins peaked on top of predicted miRNA target sites, especially on target sites of miRNAs highly expressed in HEK293 cells (*hisites*) (Fig 3a). To identify proteins that were significantly enriched around miRNA target sites, we computed an empiric p-value by comparing their enrichment around target sites with that around random target sites. Interestingly, a total of 26 out of 47 RBPs analyzed showed a significant enrichment around *hisites* (empiric p-value < 0.05; Fig 3b and S3 Fig). From these, 13 are known or predicted to interact with RISC according to STRING database [32] (S4 Fig). In some cases, the enrichment profile of the RBPs peaked on miRNA target sites. In other cases, the enrichment increased across the miRNA target site, e.g. WDR33, or was less dependent on miRNA expression, such as in the case of HuR and EWSR1 (Fig 3a and S3 Fig). Notably, the enrichment distribution around *hisites* (Fig 3b) in many cases resembles the positional correlation with AGO2 described before (Fig 2b).

To gain insight into why RBPs have a strong positional bias around *hisites*, we calculated the correlation between RBP enrichment on target sites and miRNA expression level, i.e. the sum of expressions of all the miRNA targeting them. Our results show a significant correlation between RBP enrichment on target sites and miRNA expression not only for AGO proteins (AGO1-4), but also for the proteins from the polyadenylation complex CF-Im68 and CPSF-73 and FUS (Fig 3c and S5 Fig).

For each RBP we also calculated the percentage of its clusters that overlap *hisites*. This number ranges from less than 10% to more than 40% (Fig 3d, bars), and usually is less than 5% (Fig 3d, dots). Even though the overlap was small, in most cases the association between clusters and *hisites* was significantly higher than expected by chance (permutation test p-value < 0.01; colored bars and dots). Interestingly, the combined set of all RBPs overlapped more than 77% of *hisites* (75% excluding AGO and TNRC6 proteins), which suggests that the cumulative effects of all RBPs could have a crucial role modulating miRNA function.

### PolyA complex RBPs are specifically enriched on distal *hisites*

**Fig 4:**
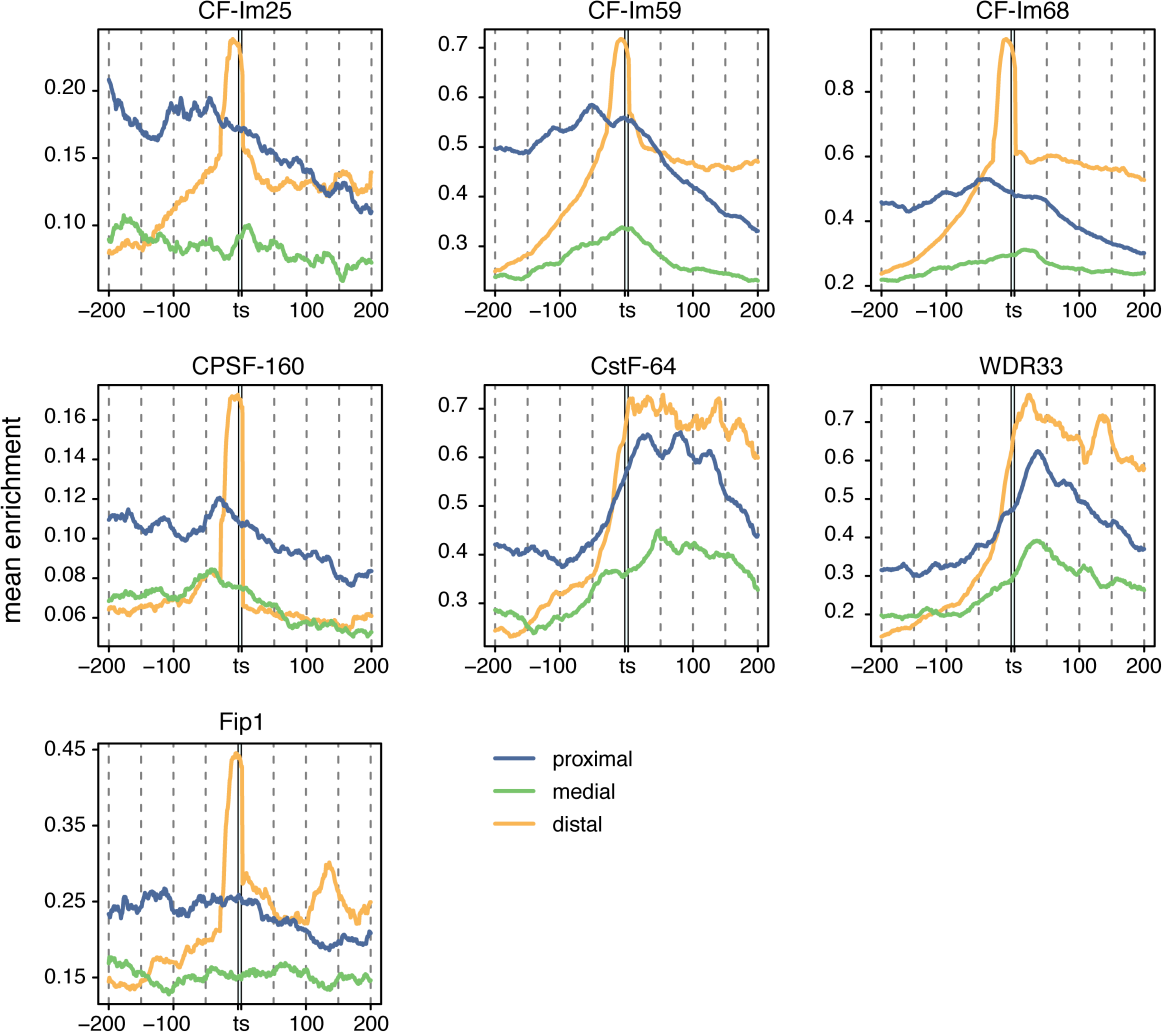
PolyA complex RBPs are enriched on distal *hisites*. CLIP enrichment of RBPs from the polyA complex around proximal (blue), medial (green) and distal (yellow) *hisites*. All the RBPs show a higher enrichment on distal *hisites* compared to proximal and medial *hisites*. These binding profiles can be grouped in two categories. In the first group, which includes CF-Im25, CF-Im59, CF-Im68, CPSF-160 and Fip1, the profiles are similar to that of AGO2. In the second set, which includes CstF-64 and WDR33, the enrichment changes from low to high across *hisites*.

Several components of the polyadenylation complex, including the cleavage factors CF-Im25, CF-Im59 and CF-Im68, showed a strong enrichment on *hisites* (Fig 3b) and an overlap with *hisites* comparable to that of AGO proteins (Fig 3d). We analyzed their enrichment on *hisites* according to their location on the 3’UTRs. Each 3’UTR was divided in three equally sized regions and *hisites* in these regions were classified according to their location as proximal, medial and distal. Our hypothesis was that if this binding was specific, RBPs from the polyadenylation complex should be more enriched on target sites at the end of the 3’UTR. As expected, we observed a strong enrichment on distal *hisites* for most of these proteins (Fig 4). Two distinct types of profiles were predominant. The cleavage factors CPSF-160 and Fip1 peaked exactly on the target site region in a similar way to AGO2 (Fig 3a), although their enrichment on *hisites* was much lower. Alternatively, CstF-64 and WDR33 displayed a change from low enrichment before the target site to high enrichment after the target site. These two different types of binding profiles around *hisites* may reflect the different kinds of interactions of these RBPs with miRNAs or the miRNA machinery.

### RBPs bind in regulatory hotspots containing miRNA target sites and AREs

To evaluate the impact of all RBPs together, we looked at their binding in non-overlapping 50 nt windows across 3’UTRs. In order to discard weak binding sites, we considered only significant clusters with a positive enrichment relative to RNA-seq data. The list of all the windows containing RBP binding sites mapped on them can be found in the S2 Appendix. We observed that the windows with more than 3 RBPs binding were more frequent than expected if RBPs would bind independently (S6 Fig). We also observed that windows containing more RBPs displayed a stronger positional bias towards 3’UTR edges, similar to that of miRNA target sites (S7 Fig).

**Fig 5:**
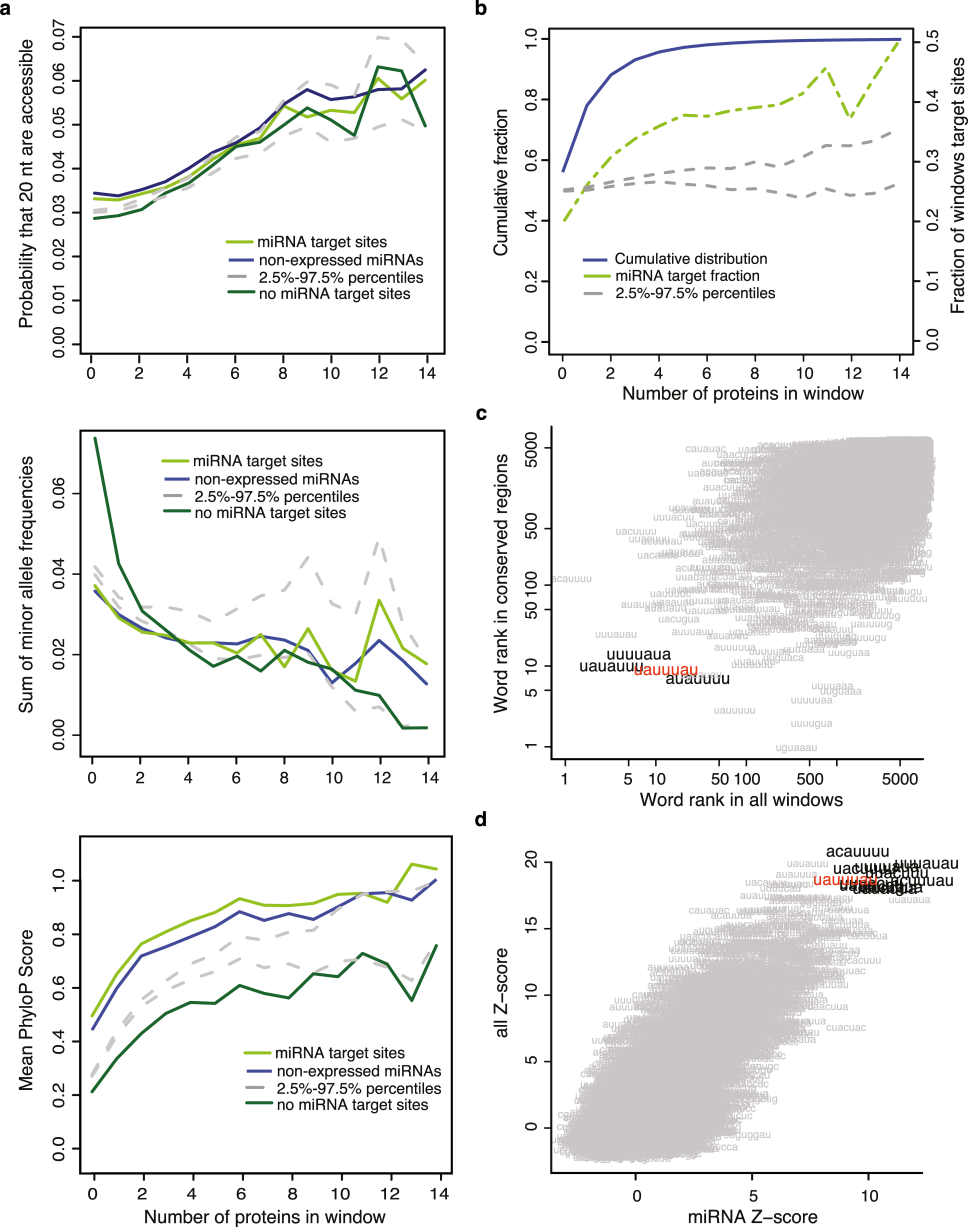
Hotspot sequences are highly conserved, accessible and contain AU-rich elements. **a)** Relation between accessibility (top), sum of minor allele frequencies (middle), and conservation (bottom) with the number of RBPs in a window. The colored lines show the behavior of windows that overlap expressed miRNAs in HEK293 (light green), non-expressed miRNAs in HEK293 (blue) or that do not overlap miRNA target sites (dark green). The grey dashed lines mark the 2.5% and 97.5% percentiles of the distribution obtained by generating 100 random miRNA datasets. The presence of miRNA target sites in the windows does not affect the correlation observed between accessibility and sum of minor allele frequencies. In contrast, windows that overlap miRNA target sites (both expressed and non-expressed) show higher conservation regardless of the amount of RBPs that bind in the window. **b)** Cumulative distribution function showing the fraction of windows (y-axis) bound by a number of RBPs (x-axis) for all windows (blue), windows containing miRNA target sites (dashed green line) and random miRNA target sites (grey lines). **c)** Scatter plot showing the correlation between the ranks of the 7-mers identified using cWords [40] ordering the windows according to mean phyloP scores (y-axis) or the number of RBPs in a window (x-axis). Highlighted are the words that are in the top-20 in both analyses. The identified ARE UAUUUAU is highlighted in red. **d)** Scatter plot showing the correlation between the z-scores of the 7-mers identified ordering the windows according to the number of RBPs in all windows (y-axis) or only in windows containing miRNA target sites (x-axis). The words with the highest z-scores in both datasets (cut offs 17.5 and 8 for all and miRNA-containing windows respectively) are highlighted. In red is highlighted the ARE UAUUUAU.

One of the main characteristics of hotspots is that they are more accessible than windows with less RBPs binding on them (Fig 5a top; spearman correlation coefficient rho 0.14, 0.14 and 0.15 for windows overlapping expressed miRNAs, non-expressed miRNAs and not overlapping miRNAs respectively; p-value < 2.2e-16 in all cases). However, if RBP hotspots are functional regulatory elements in 3’UTRs, we expect them to have some features common to other known functional elements such as a higher conservation relative to its surrounding area. For each of the 50nt windows we measured their average conservation using phyloP scores [39] and their SNP density. As seen in Fig 5a, we observed a significant correlation between the amount of RBPs binding in a window and its average conservation using phyloP scores (rho 0.19, 0.20 and 0.20 for windows overlapping expressed miRNAs, non-expressed miRNAs and not overlapping miRNAs respectively; p-value < 2.2e-16 in all cases). As expected, the conservation on average was higher for hotspots overlapping miRNA target sites, which are often highly conserved across species. Additionally, we observed that the number of RBPs binding in a window had a modest but significant negative correlation with the sum of minor allele frequencies (rho = -0.04, -0.05 and -0.03 for windows overlapping expressed miRNAs, non-expressed miRNAs and not overlapping miRNAs respectively; p-value < 2.2e-16 in all cases). This reflects both a lower frequency of SNPs and that the SNPs in the window are less frequent in the population. Together, these results indicate that RBP hotspots are functional regulatory elements under negative purifying selection.

Considering the dependencies among RBP binding sites, we decided to define hotspots in 3’UTRs as windows containing at least 5 RBPs. Some examples of RBP binding in 3’UTR hotspots can be seen in S8 Fig and S9 Fig. Using this definition, approximately 4% of all windows are classified as hotspots, whereas 56% of them are not bound by any RBPs (Fig 5b). We noticed that the number of RBPs binding in a window is positively correlated with U-content (r = 0.21, p-value < 2.2e-16) and negatively correlated with G-content (r = -0.2; p-value < 2.2e-16) (S10 Fig). Additionally, windows targeted by several RBPs have much higher sequence accessibility, measured as the probability that at least 20 consecutive nucleotides are unpaired (Fig 5a). This result is consistent with the fact that hotspots are more accessible regions, which favors the binding of multiple RBPs.

We used cWords [40] to identify motifs enriched in hotspots. We identified several AREs, including UAUUUAU, among the top 20 ranked words enriched both in hotspots and in conserved regions (Fig 5c). The core ARE element AUUUA was enriched in hotspots as well, although its frequency does not increase linearly with hotspot size (S10 Fig). We also noticed that the words enriched in hotspots overlapping miRNA target sites are very similar to those found in all hotspots (Fig 5d, S4 Table). Notably, we found an almost complete G-depletion in the top 100 words enriched in hotspots, which is consisted with the observation that hotspots have higher accessibility.

### RBP hotspots are not CLIP artifacts

A previous study concluded that many regions found to be targeted by several different RBPs in CLIP-seq experiments are artifacts caused by biases in the experimental technique [30]. To understand if the hotspots are a result of CLIP background, we investigated the overlap between the hotspot regions identified here and a set of binding sites obtained from a GFP PAR-CLIP experiment [30]. This experiment contained 3 datasets belonging to protein-RNA complexes with different molecular weight, which we analyzed using the same pipeline described before and pooled together. We identified 11323 significant GFP binding sites, i.e. background clusters. It has to be noted that our mapping pipeline discarded most of the data as insignificant, which is consistent with the expectation that there should be very few genuine binding sites (S5 Table).

The center of the significant background clusters with positive enrichment over RNA-seq were extracted and overlapped with the previously identified windows in 3’UTRs. 7% of background clusters (837 sites) overlapped with previously identified hotspots containing 5 or more RBPs. Thus, only 5% of hotspots overlapped background clusters. To validate that these background clusters were not biasing the results, we discarded all the windows containing GFP sites and repeated the analysis in Fig 5. This filtered dataset recapitulated the results obtained previously (S11 Fig). Together, these results support the idea that RBP hotspots are regulatory 3’UTR elements and not CLIP artifacts.

### RBP binding in hotspots influences mRNA stability

**Fig 6:**
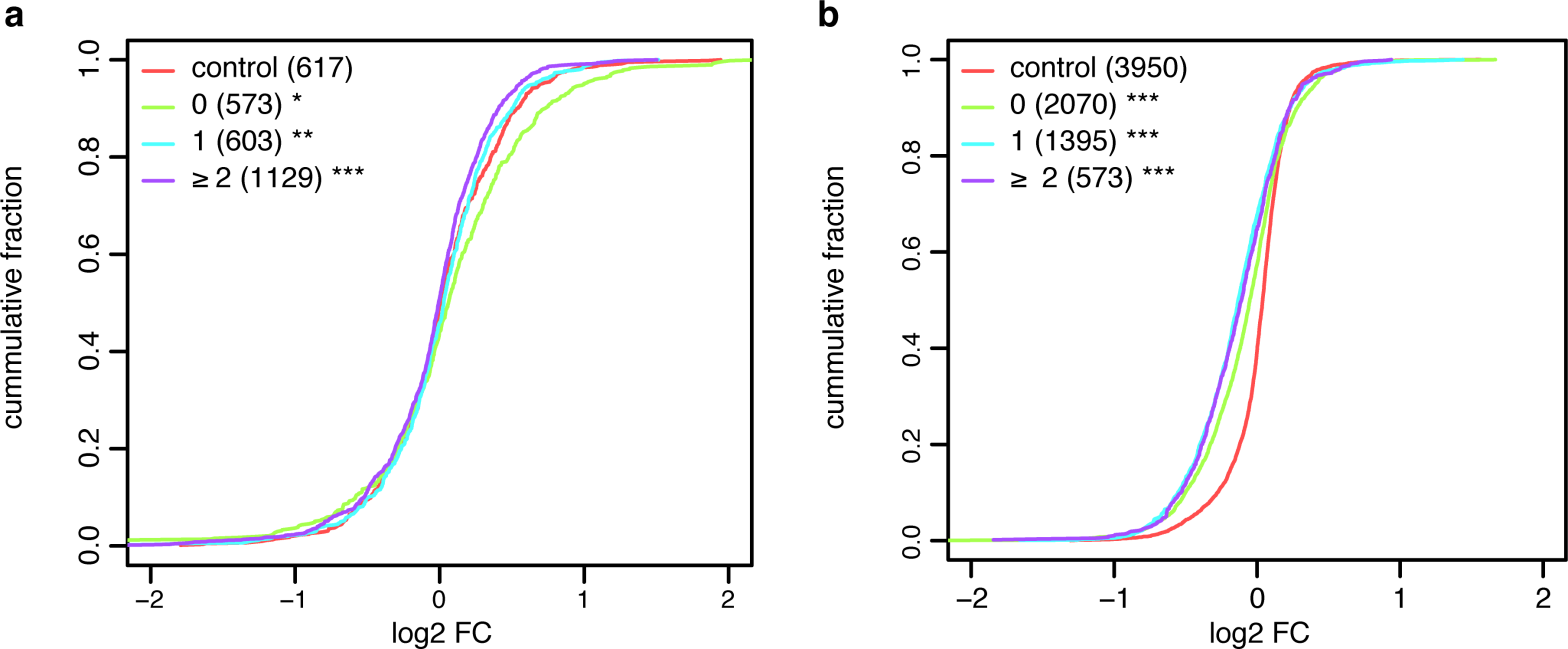
RBPs compete with each other and with miRNAs for binding on hotspots. Cumulative fraction plot showing the effect of having 0, 1 or 2 or more **a)** *hisites* **b)** HuR binding sites overlapping RBP hotspots. As an additional control genes lacking **a)** *hisites* and **b)** HuR binding sites are shown. The x-axis shows the distribution of log2FC upon **a)** AGO2 or **b)** HuR KD. The y-axis shows the cumulative fraction of transcripts. *, ** and *** denote a p-value < 0.05, 0.01 and 0.001 respectively. The sets containing 1 or more sites overlapping hotspots are compared to set without sites overlapping hotspots. The set without sites overlapping hotspots is compared to the control set.

We have observed that hotspots are more conserved, more accessible, and enriched by miRNA target sites and AREs, including UAUUUAU, which has been associated with stronger miRNA effect and mRNA stabilizing effect [3,41]. Hence, we set out to assess the effect of RBP hotspots on *hisites* using previously published AGO2 KD microarray data [42]. For each transcript, we defined a new set of 50 nt windows centered on *hisites* and measured the effect of the presence of a hotspot (excluding AGO2 when defining the hotspots) overlapping 1, or 2 or more *hisites* in a transcript upon AGO2 KD. As a control, we used two sets of transcripts, one where all the *hisites* were in windows containing 2 or less RBPs and another one in which transcripts contained no *hisites* at all. By calculating the cumulative fractions of fold expression changes of transcripts upon AGO2 KD, we found that the presence of a hotspot overlapping *hisites* in a transcript prevents its upregulation upon AGO2 KD (two tailed KS test p-value = 0.0017 and 6.9e-08 for 1 and 2 or more target sites blocked compared to genes without hotspots on *hisites* respectively) (Fig 6a). This result cannot be explained by differences in 3’UTR length, number of *hisites* in 3’UTRs, or expression biases across categories (S12 Fig). Thus, it suggests that RBP hotspots can prevent the binding of RISC on miRNA target sites.

Additionally, we explored the function of hotspots overlapping the binding sites of other RBPs using published KD data. Similarly to the previous analysis, we defined hotspots centered on significant HuR clusters identified with CLIP data and measured the effect of the presence of a hotspot (without considering HuR) overlapping 1, or 2 or more HuR binding sites upon HuR KD [16]. As a control, we used transcripts where all the HuR binding sites were located in windows containing 2 or less RBPs (not counting HuR), or not bound by HuR. As expected, upon HuR KD, transcripts containing HuR binding sites show lower expression compared to transcripts not bound by HuR (Fig 6b, two-tailed KS test p-value < 0.001 for transcripts with 1 and 2 or more HuR sites blocked compared to genes without hotspots on HuR sites). We also observed a small but significant difference between transcripts containing hotspots overlapping HuR binding sites and those that do not have them (KS test p-value < 0.001 in both cases). In this case, transcripts containing HuR sites overlapping hotspots show a decreased expression upon HuR KD than transcripts containing HuR sites outside hotspots. This result suggests that upon HuR KD other RBPs with a negative effect on mRNA stability bind in those locations and thus promote mRNA downregulation. Accordingly, we found that 55% of the hotspots overlapping HuR sites contained AGO proteins, TNRC6 proteins, AUF1 or TTP, which are all known to be involved in promoting mRNA decay. Similar results were observed when analyzing the effect of hotspots overlapping AUF1 and TTP binding sites (S13 Fig). Taken together, these results show that changing RBP concentration within cells may affect their binding on mRNAs and modify their post-transcriptional regulation.

### RBP hotspots define a post-transcriptional regulatory network

In order to understand the biological function of RBP hotspots transcriptome-wide, we sought to characterize the transcripts containing hotspots. Transcripts with hotspots possess some features that suggest that they are under strong post-transcriptional regulation, as they have longer 3’UTRs (spearman correlation coefficient rho = 0.17; p-value = 1.2 e-60) while keeping approximately the same density of miRNA target sites (S14 Fig). Furthermore, we also noticed that they are significantly higher expressed than transcripts without hotspots (rho = 0.3; p-value = 1e-191 S14 Fig).

We used PANTHER [43] to characterize their functions using GO-terms. The most significant molecular function terms identified were polyA RNA binding, RNA binding and nucleic acid binding (p-value < 0.001; S6 Table). Among the genes that contain these terms RNA binding proteins, splicing factors and transcription factors stand out (S15 Fig), which suggests that hotspots could be central in the regulation of both transcriptional and post-transcriptional processes.

## Discussion

In this work, we have reanalyzed a comprehensive collection of high-throughput CLIP experiments in HEK293 cells in order to better understand the complex interactions between RBPs and miRNAs in post-transcriptional regulation. Our results show that RBPs and miRNAs often bind in the same regions within 3’UTRs, which suggest that they function as regulatory hotspots that facilitate competition between the regulators. We show that these hotspots function in an analogous manner to promoter regions in accessible chromatin regions, and the RNA fate depends on which of the regulators bind to the mRNA. In turn, this regulation would also depend on external cues or post-translational modifications that modulate the relative concentration of these factors or their affinity to mRNA. Interestingly, RBP hotspots are enriched in transcripts involved in transcriptional and post-transcriptional regulation, such as RNA binding proteins, splicing factors, transcription factors and translation factors (S15 Fig), thus suggesting that these regulatory hotspots play a role in auto-regulatory networks of regulators previously reviewed [44]. This result is also in agreement with recent findings that show that RNPs tend to regulate the mRNAs of other RNPs and themselves thus creating auto-regulatory networks in *Drosophila* [45].

We have used positional correlations to assess the interactions between RBPs assuming that RBPs that bind in the same location may interact. Surprisingly, we found that most RBPs (69% of all RBP pairs analyzed) tend to have overlapping binding sites (Fig 2a and S2 Fig). Using this approach we confirmed some known positional correlations, such as those among polyA complex proteins [46], the IMP and the AGO proteins [36], the FET family proteins (TAF15, FUS, EWSR1) [47], and the snoRNA processing proteins FBP, NOP56 and NOP58 [48]. Moreover, we found correlations that were previously unknown. Some of these can be explained by the similarity in binding motifs, such as those among HuR, TTP and AUF1 [11,13,16]. However, the consistent correlation of all the RBPs analyzed with AGO2 had not been previously described in literature. Additionally, the finding that many of these RBPs are also enriched on *hisites* further supports the positional correlations. It has to be noted that these miRNA target site predictions are independent of the CLIP data [49], which speaks against these overlaps being an artifact of the CLIP protocol.

One intriguing question is why the RBPs bind on miRNA target sites. If RISC directly interacts at miRNA target sites with a particular RBP, it would be expected that CLIP enrichment covaries with the expression of the miRNA that targets it. Yet, in most cases we did not find a clear correlation (S5 Fig) and thereby direct interaction is probably not the general mechanism to explain RBP enrichment at miRNA target sites. Nevertheless, we found a positive correlation for several proteins from the polyadenylation complex although it is only significant for CPSF-73 and CF-Im68 (Fig 3c). We found that many proteins from the polyA complex bind at *hisites*. As expected, we observed a stronger enrichment of the RBPs from the polyA complex on distal *hisites* compared to proximal or medial (Fig 4). Interestingly, we observed two clearly different types of binding profiles around *hisites*: one for the cleavage factors, Fip1 and CPSF-160, which show a strong enrichment specifically on distal *hisites*; and another one for WDR33 and CstF-64, which show an increase in the enrichment specifically after *hisites*. Our data merely shows that miRNAs and the RBPs from the polyA complex bind in the same location, which is likely the result of the presence of both sets of regulators in hotspot regions, which are more accessible, and thus preferred for both purposes. One may speculate that the strong enrichment of polyA complex RBPs on *hisites* could indicate some kind of functional interaction between these two pathways. However, the nature of these interactions cannot be explored using computational methods and additional experiments would be required to investigate it.

We have also shown that RBP binding sites cluster in regulatory hotspots in 3’UTRs. These hotspots are more frequent than expected if RBPs would bind independently (S6 Fig) and are significantly enriched on predicted miRNA target sites (Fig 5b). Furthermore, they are AU-rich (S10 Fig) and contain AREs, which are both more conserved and overrepresented (Fig 5c). AREs and AU-rich context of miRNA targets have previously been associated with effective miRNA target sites [3,41,50] and are known to be targeted by many RBPs both with stabilizing and destabilizing functions. These results, together with the high accessibility of the RBP hotspots, could explain the large number of RBPs binding in the regions and their role as regulatory elements in 3’UTRs. It has to be noted that several of the RBPs analyzed in this paper are known to bind A-, U- or AU-rich motifs. Some of these RBPs, such as HuR, TTP and AUF1, can bind AREs. However, many others bind completely different motifs, such as the RBPs from the FET family, which bind AU-rich stem loops, and the proteins from the polyadenylation complex among others [11,13,16,36,46,47,51]. Besides, CLIP crosslinking introduces a U-bias, either by the use of UV-C radiation in HITS-CLIP and iCLIP [52] or by the incorporation of 4-thiouridine in the RNA to cross-link the RBPs in PAR-CLIP [36]. These two factors may explain a part of the U-richness observed in the hotspots, as shown by a sequence composition analysis of the significant clusters in 3’UTRs of the individual RBPs (S16 Fig).

Finally, we have shown that RBP hotspots regulate miRNA target site accessibility and could favor the competition between miRNAs and RBPs in 3’UTRs. Upon AGO2 KD, transcripts containing miRNA target sites in hotspots do not show significant increased expression levels, which suggests that these target sites were protected by RBPs binding in the same hotspots (Fig 6a). Interestingly, the opposite effect was found upon KD of HuR, AUF1 and TTP. Upon KD of these RBPs, transcripts containing their binding sites in hotspots show a stronger reduction in expression levels than those that have their binding sites isolated. A high fraction of those hotspots contain AGO2 or other down regulatory RBPs, which suggests that by removing the RBPs, other ones bind and affect mRNA stability (Fig 6b and S13 Fig). These results are in agreement with recently reported findings that show that the presence of RBP binding sites of overlapping PUM1/2 or HuR binding sites reduce their impact on mRNA stability [26].

A previous study concluded that many regions found to be targeted by several different RBPs in CLIP-seq experiments are artifacts caused by biases in the experimental technique [30]. As a result, these regions have been excluded from previous works analyzing the combined effect of RBPs and miRNAs in post-transcriptional regulation [26]. In our analysis, we observed that the number of RBPs that target a hotspot weakly correlates with mRNA expression (S14 Fig) regardless of the use of mFDR and RNA-seq normalization of CLIP data. This bias probably hinders the identification of hotspots in lowly expressed genes, but it is unlikely that this is the reason why we observe hotspots in 3’UTRs. To validate that these regions are truly regulatory elements, we have analyzed the 3 GFP PAR-CLIP experiments used to identify CLIP artifacts [30] and studied their overlap with hotspots. Using our pipeline, only 2% and 9% of the original GFP libraries can be confidently mapped to the genome (S5 Table). These results confirm the stringency of our pipeline and give extra evidence that the processing pipeline discards most of the spurious CLIP binding artifacts. Besides, additional analyses of this dataset further support that the identified regulatory hotspots are not the result of background CLIP binding sites. Firstly, it was described that background reads, i.e. the reads that appear in multiple datasets derived from a CLIP experiment of a protein that does not bind RNA [30], are G-rich. In contrast, our RBP hotspots are characterized by a general G-depletion and are AU-rich (S10 Fig). Secondly, the CLIP enrichment of RBPs is in most cases not different inside or outside hotpot regions. If CLIP binding in hotspot regions would be spurious, we would expect a consistently lower CLIP enrichment of all RBPs in these regions. However, for many of the RBPs analyzed the number of RBPs in a window does not affect the distribution of their enrichment values (S17 Fig). Thirdly, the regulatory hotspots that we identify are experiencing increased selective pressure, as shown by the higher PhyloP scores and lower SNP frequencies (Fig 5a). The correlation between conservation and number of RBPs in a window is observed for all windows regardless of their overlap with miRNA target sites, which indicates that the increased selective pressure happens due to the preservation of binding sites for more RBPs. Moreover, it supports the conclusion that these regions are indeed functional regulatory elements. Fourthly, we show that hotspots more often coincide with miRNA target sites, which are independent of CLIP-seq data. In conjunction, we see a significant functional effect of hotspots in the regulation of sequence accessibility both using KD data from AGO2 and other RBPs (Fig 6 and S13 Fig). Taken together, we believe that our stringent pipeline for the processing of the datasets, which includes duplicate removal, quality score aware mapping of reads, peak calling of clusters in transcripts, and normalization by gene expression, removes or diminishes the importance of most of the reads that were shown to result in background when a less stringent data pipeline was used [30] and thus allow us to identify truly regulatory regions targeted by several RBPs. Accordingly, only 5% of our regulatory hotspots, i.e. windows containing 5 or more different RBPs, overlap background sites identified by analyzing the GFP CLIP sites identified with our pipeline. Removal of these windows from our dataset did not alter the characteristics of the identified hotspots (S11 Fig), which confirms our observation that RBP hotspots are not CLIP artifacts.

Many studies have previously investigated the interaction between miRNAs and RBPs using both experimental and computational methods [14,25,26,53,54]. Both competition and collaboration between miRNAs and RBPs have been described, but these interactions have been often portrayed as isolated events rather than a general mechanism in post-transcriptional regulation. In this work, we have shown that the overlap between miRNA target sites and RBPs is very extensive, with more than 75% of all *hisites* targeted by one or more of the RBPs analyzed (excluding AGO and TNRC6 proteins), thus suggesting that RBP hotspots play a major role in miRNA regulation and post-transcriptional regulation.

Taken together, our analyses suggest that post-transcriptional regulation often happens in hotspots where several trans-acting factors bind and may compete and cooperate for regulating mRNAs. This organization thus facilitates fast changes on mRNA expression induced as a response to external cues and facilitate cell adaptation to environment changes.

## Materials and methods

### Mapping and processing of CLIP and RNA-seq datasets

110 CLIP (including CLIP-seq and PAR-CLIP) and 3 RNA-seq datasets were downloaded from GEO database [55]. The Sequence Read Archive (SRA) accession numbers of all the datasets analyzed can be found in S5 Table.

Reads from all the experiments were preprocessed using custom python scripts. First, reads were trimmed to remove low quality scores and 3’ adapter sequences (only CLIP datasets). Next, we removed duplicates by collapsing all identical reads. This step was performed instead of collapsing reads that map to identical locations to keep fragments that contain different mutations as a result of cross-linking and that represent true crosslink events that otherwise would be discarded. After these steps, all reads longer than 19 nucleotides were further analyzed. This minimum length was set to minimize the amount of incorrectly mapped reads that could come from contamination [56]. Reads were mapped to the human genome (hg19) using BWA-PSSM with parameters -n 0.04 -l 1024 -m 400 -P 0.5 [35]. Then, all unmapped reads were then mapped to an exon-junction index containing all annotated unique exon-junctions from human Ensembl70 transcripts [57]. Only reads mapped at any of the steps with a posterior probability > 0.99 were considered for further analysis. For PAR-CLIP datasets, we used a custom matrix for scoring T to C mismatches assuming a 12.5% T to C conversion rate.

Datasets for the same proteins were joined into a single dataset and analyzed together. Additionally, we also pooled the datasets of CstF-64 and CstF-64τ and FXR1 and FXR2. Reads were clustered according to their genomic positions, requiring that at least 1 nucleotide overlap. Significant clusters were calculated using Pyicos [38], using the exons from the longest protein coding transcript for calculating the randomizations. Only clusters with a false discovery rate (FDR) < 0.01 were considered for further analysis. The RNA-seq datasets were also joined and used together in further experiments. The statistics summarizing the preprocessing steps, mapping, clustering and peak calling can be found in S5 Table. Additional analysis performed to validate the processing pipeline can be found in the S1 Appendix. The pipeline used for preprocessing and mapping of the data is publicly available on GitHub under an Open Source license (https://github.com/simras/CLAP).

### GO-term enrichment analysis

We obtained the significantly overrepresented biological process GO-terms associated with the RBPs included in the analysis using the gene enrichment analysis method performed by PANTHER [58]. The clustering and visualization of enriched GO-terms was done using REVIGO (http://revigo.irb.hr/) [59].

### Gene expression

For each 3’UTRs we calculated *m*_*k*_, the average number of RNA-seq base calls per nucleotide and then we normalized to *M*, the total amount of mapped RNA-seq reads in the experiment as follows

$$m_{k}=\sum_{j=1}^{l_{k}}\frac{r_{j}}{M\cdot l_{k}}$$

where *l*_*k*_ is the length of the 3’UTR for gene *k*, and *r*_*j*_ is the count of RNA-seq reads in position *j*.

### CLIP enrichment in 3’UTRs

For each transcript, we built a single-nucleotide resolution profile of the RBP binding sites, i.e. significant clusters with an FDR < 0.01 after peak calling, normalized to RNA-seq. The enrichment *e* of CLIP in a position *i* of a particular 3’UTR *k* is calculated as

$$e_{i,k}=\frac{c_{i}}{m_{k}\cdot N}$$

where *c*_*i*_ is the count of clip reads in position *i*, *N* is the total amount of uniquely mapped CLIP reads, and *m*_*k*_ is the average gene expression as defined above.

### Selection of miRNA target sites

Good miRNA target site predictions for conserved and non-conserved miRNAs were downloaded from microRNA.org (http://www.microRNA.org; August 2010 release) [49]. From this set, we selected only target sites containing at least a 6-mer seed site, and selected targets that belonged to miRNAs expressed in HEK293.

We used small RNA-seq data (GSM1279922) [60] to estimate the expression levels of each miRNA. First, we selected from the dataset reads that were 15-27nt long, which corresponds to the length range of mature miRNAs. Next, we mapped the RNA-seq to a set of non-redundant human miRNA sequences downloaded from miRBase [8] using BWA-PSSM [35]. The expression of each miRNA was defined as the number of reads mapping to its mature miRNA sequence. We defined as expressed miRNAs only the top 20% of the mature miRNAs (155 miRNAs; minimum amount of mapped reads mapped 367). All the other miRNAs not included in this set are regarded as non-expressed miRNAs in HEK293 cells.

For each of the target sites, we selected the set of targets that overlapped Ensembl70 [57] transcripts expressed in HEK293 cells and defined a set of non-overlapping target sites. To define which target sites to keep, we overlapped the seed sites of their target sites and kept the one targeted by the most highly expressed miRNA. If several miRNAs shared the target site, we added their expression. Finally, we kept only target sites that contained a 6-mer seed site in the selected transcript. The number of target sites kept at each step of the processing is summarized in S7 Table.

For some of the analyses we divided target sites according to their total expression, i.e. the sum of expressions of miRNAs targeting the same site, in three equally sized groups: highly expressed (*hisites*), moderately expressed and lowly expressed.

### Random miRNA target sites

To measure the significance of our results, we created 100 random sets of miRNA target sites containing as many target sites as the original set preserving their distribution along 3’UTRs. We divided the set of expressed genes with predicted miRNA target sites into 30 equal size groups with similar 3’UTR lengths. Then, for each target site in a particular 3’UTR, we assigned it to another of the 3’UTRs in the set. In case that the length of the new 3’UTR was different from that of the original 3’UTR, the relative coordinates of the target site were calculated so that it would have the same relative position within the 3’UTR in relation to its length. This procedure preserved the characteristic positional distribution of miRNA target sites along 3’UTRs.

### Mapping CLIP clusters on 3’UTRs

The significant CLIP clusters for each of the RBPs were overlapped with the genes from Ensembl70 [57] annotation using fjoin [61]. Only the longest protein-coding transcript for a gene was considered. If a cluster would overlap the CDS and a UTR region, the UTR annotation was assigned.

### 3’UTR positional data distribution

We analyzed the positional distribution of data across 3’UTRs of expressed genes (RNA-seq coverage > = 50%) and around *hisites*. Each 3’UTR was divided in 50 equally sized bins. For each bin, the mean value per nucleotide was calculated and then averaged across all expressed genes. In the case of CLIP data, the position of significant CLIP clusters (FDR < 0.01) was used to draw the profiles. In the case of hotspots, the position of the 50nt windows containing n (n = 1,2,…31) RBPs mapped on them was used. For miRNA target sites, the position of the target seeds in 3’UTRs was used.

### Positional correlation analysis

To find the positional correlation between the binding of two different proteins, we calculated the Pearson correlation between the enrichment values along a 3’UTR. If the value at position *i* is called *x*_*i*_ for one RBP and *y*_*i*+*d*_ for the other RBP binding a distance *d* from the first, the Pearson correlation was calculated with fixed *d* over all positions *i*, in the interval from 1 to *l* − *d*, where *l* is the length of the 3’UTR (for negative *d*, the interval is from 1 − *d* to *l*). This was done for all values of *d* from -200 to 200. For each *d*, the correlation values were averaged over 3’UTRs. UTRs shorter than 400 nt were discarded.

The fluctuations of the correlation coefficients are heavily dependent on the number of CLIP sites. To estimate the background distribution, we shuffled the CLIP data in a way that preserved the clustering of tags. Clusters were defined as contiguous regions in which the enrichment value was above 10^−6^. The clusters identified in a sequence were moved to a random location in the sequence while ensuring at least one position in between clusters. After shuffling all sequences, positional correlations were calculated as above. This was repeated 100 times and for each *d*, and the mean and standard deviation of the 100 values obtained in the shufflings were calculated. Using these estimates, the z-score was calculated for the unshuffled data. In Fig 2a, the distribution of all z-scores calculated was considered and divided in 1000 quantiles. Each quantile was assigned a color from the scale, ranging from dark blue to red as shown. In Fig 2b, z-scores were row-normalized and assigned a color using the same procedure as described above.

### Hotspot identification

To identify hotspots we divided the 3’UTRs of expressed genes (at least 50% RNA-seq coverage in the 3’UTR of the longest protein-coding transcript) in non-overlapping windows of 50 nt. We overlapped the center of the RBP CLIP significant clusters with them and assigned each cluster to a single window. Only clusters with a positive enrichment over RNA-seq were considered. We also uniquely assigned each miRNA target site of expressed miRNAs in HEK293 cells to a window if the overlap between the seed site and the window was bigger than 5. Otherwise, the miRNA target sites were discarded.

### Simulation of RBP binding site distribution on hotspots

We simulated 10000 times the distribution of hotspot sizes by randomly sampling the binding location of the proteins assuming a uniform distribution of the RBPs in them. We considered the total amount of windows in which we observe significant clusters of each RBP and the total amount of windows in 3’UTRs (S7 Table). The size distribution of hotspots from simulated and real data can be seen in S6 Fig.

### Analysis of hotspot conservation

PhyloP scores [39] calculated from 100 vertebrate genome alignments (including hg19 human genome assembly) were downloaded from UCSC genome browser. For each of the 50nt non-overlapping windows, we calculated the mean phyloP score across the window, discarding regions that were not present in any of the other species.

### Motif analysis

Word enrichment analyses were done using cWords [40]. The input data sets were made using the 3’UTR window data described above. For each window, we extracted its sequence and associated it to the number of RBPs binding in it. Using this method we defined two datasets: one containing all windows and another one containing only those overlapping target sites for expressed miRNAs.

In the first analysis, windows were ranked using the amount of RBPs binding in them. Thus, the resulting words were differentially enriched in windows according to the number of RBPs binding in them. In the second analysis, we ranked the windows using their mean phyloP score.

### RNA secondary structure accessibility of 3’UTR windows

We used RNAplFold [62] to calculated the sequence accessibility of the 3’UTRs. Specifically, we predicted the probability that 20 contiguous nucleotides in the sequence are unpaired using the parameters -u 20 -L 40 -W 120. The obtained accessibility values were then mapped to the 3’UTR windows and averaged across windows with the same number of RBPs binding and across windows with the same number of RBPs that overlap miRNA target sites.

### Minor allele frequency analysis

The complete data set of the 1000 genomes project containing all variants mapped to hg19 assembly [63] was downloaded. Of all the variants, we only used mutations regardless of their size and required them to be present in at least two individuals in a population of 5008. We calculated the mean of the sum of all minor alleles as 1 - major allele frequency regardless of which was the reference allele across windows as described above.

### Knockdown data analysis and processing

We downloaded the microarray data containing the expression values for AGO2 KD (GSM95818, GSM96819, GSM96816 and GSM96817) [42] and HuR KD (GSM738179, GSM738180, GSM738181, GSM738182, GSM738183) [16] from GEO database. We calculated differential expression upon AGO2 KD using the *limma* package [64] in R. We also downloaded processed data from KD experiments in AUF1 [13] and TTP [11].

### Cumulative fraction plots

We defined 50 nt windows around *hisites* (35 nt upstream of the target site 3’ end, 14 nt downstream of the target site 3’end). If the windows extended beyond transcript boundaries, we shrank them so that they would fit inside the transcript. In each of these windows, we checked the presence or absence of each of the RBPs.

For each transcript we measured the amount of *hisites* that would be free, i.e. 2 or less RBPs (excluding AGO2) would bind in the window around the *hisite*, and the amount of hisites that would be blocked, i.e. 5 or more RBPs (excluding AGO2) would bind in the window around them. We used these measurements to divide the genes according to the amount of free or blocked *hisites* they contained in 3 groups: 0, where all target sites are free; 1, where only 1 target site was blocked; 2 or more, where 2 or more target sites were blocked. As an additional control, we added the rest of genes containing no *hisites*.

For cumulative fraction plots centered on RBP binding sites, we defined 50 nt windows centered around the binding sites of the RBP of interest. The groups of transcripts used to evaluate the role of hotspots on RBP binding sites were built in an analogous manner to the one described above.

## Supporting information

S1 Appendix Additional analyses to validate the processing pipeline. (DOCX)

S2 Appendix Identified hotspots in HEK293 cells. Extended bed file containing all the 50nt 3’UTR windows containing RBP binding sites considered in this study. For each window, the genomic coordinates of the window and the list of all the RBPs binding in them, and their enrichment, is provided.
(BED)

S1 Table Description of the RBPs analyzed in this paper. (XLSX)

S2 Table GO-term enrichment analysis of the RBPs analyzed. (XLSX)

S3 Table Fraction of miRNA target sites bound by RBPs. (XLSX)

S4 Table Word enrichment analysis on windows. (XLSX)

S5 Table Summary of mapping statistics. (XLSX)

S6 Table GO-term enrichment analysis of genes containing hotspots. (XLSX)

S7 Table Summary of miRNA target site processing statistics. (XLSX)

S8 Table Window counts used in the simulation. (XLSX)

S1 Fig T to C conversions are the main type of mutations in PAR-CLIP reads. Mutation analysis of confidently mapped reads included in this study. For each library analyzed, we have classified reads according to whether there is only one read that maps to a particular genomic location (unique reads, dark grey) or multiple reads that map to the same location (duplicated reads, light grey). For each of these groups, we show in the right panel the % of reads that have no mutations, T>C conversions or other types of mutations. RNA-seq datasets and DGCR8 dataset, which are not expected to have high T>C mutation rates are highlighted in red and green respectively.
(TIF)

S2 Fig RBP subsets show strong positional correlation across 3’UTRs. Pearson correlation coefficients of cluster enrichments are calculated for each pair of proteins (purple line) in a distance from -200 to 200 nt and represented using a color scale from blue (pearson correlation coefficient = -1) to red (pearson correlation coefficient = 1). All against all positional correlations calculated in this way are summarized in the heatmap (bottom). For each RBP in a column we show the pearson correlation coefficient of the RBPs in the rows around its binding sites.
(TIF)

S3 Fig CLIP enrichment around miRNA target sites. CLIP enrichment around miRNA target sites that are highly (red), moderately (green) and lowly (blue) expressed for the other 43 RBPs analyzed. The grey and the black lines show the maximum and the minimum enrichment values for the 90% confidence intervals around random target sites.
(TIF)

S4 Fig STRING interaction network of the RBPs analyzed. In this graph RBPs are depicted as nodes. Two RBPs are connected through edges if they interact according to STRING database. The color of the edges represent the type of interactions among them, which include known interactions, predicted interactions and other types of associations.
(TIF)

S5 Fig Correlation between CLIP enrichment and miRNA expression. Scatter plots showing the relation between CLIP enrichment at miRNA target sites (y-axis) and the expression of miRNAs targeting them (x-axis). Axes are shown in log scale. For each RBP, the Pearson correlation coefficient *r* is shown. Significant correlations are marked as *, ** or *** corresponding to p-values < 0.05, < 0.01 and < 0.001 respectively.
(TIF)

S6 Fig Hotspots in 3’UTRs are less frequent than expected by chance. Hotspot size distribution in simulated data (grey lines) and real data (red line). The x-axis shows the hotspot size and the y-axis the count of windows containing hotspots with a specific amount of RBPs.
(TIF)

S7 Fig Positional distribution of windows containing a specific amount of RBPs. Each colored line shows the ratio of the amount of windows with a specific amount of RBPs in a bin relative to the average amount of windows with that specific amount of RBPs across bins. The distribution of all hotspots, i.e. windows with more than 4 RBPs, is shown with a dashed grey line. For each line, the y-axis shows the enrichment relative to the mean of the line whereas the x-axis shows the position of the bin across a length-normalized 3’UTR. For comparison, the enrichment of miRNA target sites relative to the mean number of target sites per bin is shown.
(TIF)

S8 Fig Characteristic binding of RBPs on hotspots on the plus strand. UCSC screenshots displaying the binding of several RBPs on two of the hotspots identified. For each of the RBP tracks, the height of the tracks represents the amount of CLIP reads binding in a particular location. Additionally, SNPs, miRNA target sites used in this paper and phyloP scores are shown.
(TIF)

S9 Fig Characteristic binding of RBPs on hotspots on the minus strand. UCSC screenshots displaying the binding of several RBPs on two of the hotspots identified. For each of the RBP tracks, the height of the tracks represents the amount of CLIP reads binding in a particular location. Additionally, SNPs, miRNA target sites used in this paper and phyloP scores are shown.
(TIF)

S10 Fig Hotspots are AU-rich. The fraction of A, U, C and G nucleotides in a window is represented by a red, blue, black and pink line respectively. The x-axis shows the number of RBPs in a window. The y-axis shows the fraction of nucleotides in the window. Additionally, the green line shows the fraction of the windows that contain 1 or more occurrences of the core ARE AUUUA.
(TIF)

S11 Fig Hotspot sequences that do not overlap background clusters preserve the same properties of the whole hotspot dataset. Relation between accessibility (top), sum of minor allele frequencies (middle), and conservation (bottom) with the number of RBPs in a window after excluding all the windows that overlap background GFP clusters. The lines show the behavior of windows that overlap expressed miRNAs in HEK293 (light green), non-expressed miRNAs in HEK293 (blue) or that do not overlap miRNA target sites (dark green). The grey dashed lines mark the 2.5% and 97.5% percentiles of the distribution obtained by generating 100 random miRNA datasets. The presence of miRNA target sites in the windows does not affect the correlation observed between accessibility and sum of minor allele frequencies. In contrast, windows that overlap miRNA target sites (both expressed and non-expressed) show higher conservation regardless of the amount of RBPs that bind in the window. All the features analyzed show significant correlations of similar magnitude to those observed in the whole dataset.
(TIF)

S12 Fig Relationship between expression changes upon AGO2 KD and gene features. Correlation analysis between log2 fold changes and **a)** 3’UTR length and **b)** number of target sites in the 3’UTR. Transcripts were divided in 5 equally sized groups (left plot) according to their 3’UTR length or the number of target sites and for each of the groups the cumulative distribution functions of the log2 fold changes are displayed (right plot). No significant differences are found between contiguous groups of transcripts (KS test). **c)** Comparison of log2 fold changes across expression matched gene sets (left) with 0, 1 or 2 or more *hisites* overlapping RBP hotspots (right). In the left panel, the boxplots summarize the average expression of the genes in each of the groups measured using microarray data. For each of the original datasets, 387 genes were sampled so that the expression distribution was preserved. Upon AGO2 KD, significant differences (KS test p-value < 0.01) are only observed for the set with 2 or more *hisites* covered by hotspots compared to the set with free *hisites*.
(TIF)

S13 Fig Hotspot effect on RBP binding sites. Cumulative fraction plot showing the effect of having 0, 1 or 2 or more **a)** TTP binding sites or **b)** AUF binding sites overlapping RBP hotspots. The x-axis shows the distribution of log2FC upon KD of AUF or TTP respectively, and the y-axis shows the cumulative fraction of genes. As a control, genes with **a)** no TTP and **b)** no AUF binding sites are shown. *, ** and *** denote a p-value < 0.05, 0.01 and 0.001 respectively. The sets containing 1 or more sites overlapping hotspots are compared to set without sites overlapping hotspots. The set without sites overlapping hotspots is compared to the control set.
(TIF)

S14 Fig Correlation between maximum hotspot size and transcript features. Boxplot distribution showing the correlation between the maximum hotspot size in a transcript (x-axis) and **a)** 3’UTR length, **b)** target site density and **c)** expression levels. The spearman correlation coefficient rho and the p-value of the correlation are shown.
(TIF)

S15 Fig Hotspots are overrepresented in genes with regulatory functions. Barplot distribution of the most abundant PANTHER protein classes in genes containing hotspots annotated with the significant GO terms **a)** PolyA RNA binding **b)** RNA binding and **c)** nucleic acid binding. For each of the plots, the number of genes in each of the groups is given. Only those protein classes present in at least 1% of the genes in each category are shown.
(TIF)

S16 Fig Significant CLIP clusters in 3’UTRs are AU-rich. Barplot showing the % of AU content of the significant clusters identified in 3’UTRs for all the CLIP datasets analyzed compared to general AU-content of 3’UTRs (black line; left axis). Red dots indicate log2 ratio of U vs A content for each RBP, which is higher than the bias observed in whole 3’UTRs (dashed red line; right axis). Most datasets show a higher AU bias compared to general 3’UTRs, which is mainly due to an increase proportion of Us in the sequence, probably due to a technical bias introduced by PAR-CLIP.
(TIF)

S17 Fig Relation between hotspot size and RBP enrichment. Boxplots showing the relation between the number of RBPs in a hotspot (x-axis) and RBP enrichment on hotspots (y-axis) for all RBPs analyzed. For each RBP, the Pearson correlation coefficient *r* is shown. Significant correlations are marked as *, ** or *** corresponding to p-values < 0.05, 0.01 and 0.001.
(TIF)
