# Supplementary material for: Highly accessible AU-rich regions in 3’ untranslated regions are hotspots for binding of regulatory factors
Source: PLoS Comput Biol. 2017 Apr 14;13(4):e1005460. doi: 10.1371/journal.pcbi.1005460 (PMC5409497; doi:10.1371/journal.pcbi.1005460)
Supplement: S1 Appendix — (DOCX) [file pcbi.1005460.s001.docx]

**Appendix 1. Additional analyses to validate the processing pipeline.**

To validate the performance of the mapping and processing pipeline presented in this paper, we have performed additional analyses on the well-characterized set of polyA complex RBP to recapitulate previous findings. We used the PAR-CLIP datasets of polyA-complex proteins CstF-64, CstF-64tau, Fip1, CPSF−160, CPSF−100, CPSF−73, CPSF−30, CFIm68, CFIm59, CF-Im25[1] and WDR33[2] used in the paper

First, we looked at the positional distribution of significant CLIP clusters of these RBPs on expressed mRNAs as defined in the main paper. PAR-CLIP enrichment was calculated by correcting for expression measured by RNA-seq as described in the methods section. Mean CLIP cluster enrichment was calculated in fixed number of bins over the respective genomic regions (5’UTRs, CDS and 3’UTRs) and stitched together; more details are given in methods. As expected, CstF-64 and WDR33 are strongly enriched in 3’UTRs compared to the CDS and 5’UTR regions (Figure 1).

**Figure 1**: Profile of mean PAR-CLIP enrichment of WDR33 (A) and CstF-64 (B), binned along the 5’UTR, CDS and 3’UTR region of 12567 genes, where more than 50 % of the longest transcript is covered by RNA-seq.

To validate the previously described positional distribution of these RBPs relative to 3’ends, we analyzed in detail their enrichment in the last 300 nt of the 3’UTRs of annotated mRNAs. As seen in Figure 2, WDR33 and CstF-64 are quite specifically enriched in the immediate vicinity of the annotated cleavage sites, which recapitulates the previously published findings[1,2]. PAR-CLIP enrichment profiles of CFIm68, CFIm59, CFIm25 and Fip1 peak in same areas relative to cleavage site and have similar signal to background ratios as in Fig 3A of [1].

**Figure 2**: Mean enrichment profile for several of the RBPs from the polyA complex in the last 300 nt. From 3’UTRs

To further investigate the binding preferences of these RBPs, we performed motif analysis of the sequences overlapped by CLIP-reads of the above RBPs using cWords [3]. For each of the RBPs, significant CLIP clusters were ranked by the number of reads at the cluster summit. Only the 15000 most enriched clusters in 3’UTRs for each RBP were considered in the analyses. The analysis was restricted to hexamers using a 0-order Markov Model. Resulting z-scores and ranks were downloaded from the webserver and plots prepared in R [4]. As seen in Figure 3, the strong PAS motif AAUAAA was the most enriched motif in all but two of the datasets analyzed (CPSF-30 and CPSF-73). Data quality may be somewhat low for CPSF-proteins as these datasets only exhibit weak binding specificity in vicinity of cleavage sites (see Fig 3A in [1]) and PAS does not have so high z-scores in the motif analyses that we have performed.

**Figure 3**: Overrepresentation (z-score from cWords of y-axis) of the strong polyA signal AAUAAA in the polyA RBP CLIP datasets analyzed in the paper. (***) indicates if the PAS ranked as word number 1.

The full set of enriched words for WDR33, CstF-64 and Fip1 can be seen in Figure 4. In the figure, each dot represents a word, which are colored according to word similarity. miRNA seed regions of target sites identified are annotated on the plots by triangles. As can be seen in the Figure, not only the canonical PAS signal AAUAAA but also the weak PAS AUUAAA, and UG-rich motifs are found highly over-represented.

In conclusion, we consider that our analyses reconstitute known and expected relationships among the RBPs from the polyA complex well and support the validity of the analysis pipeline used in the paper.


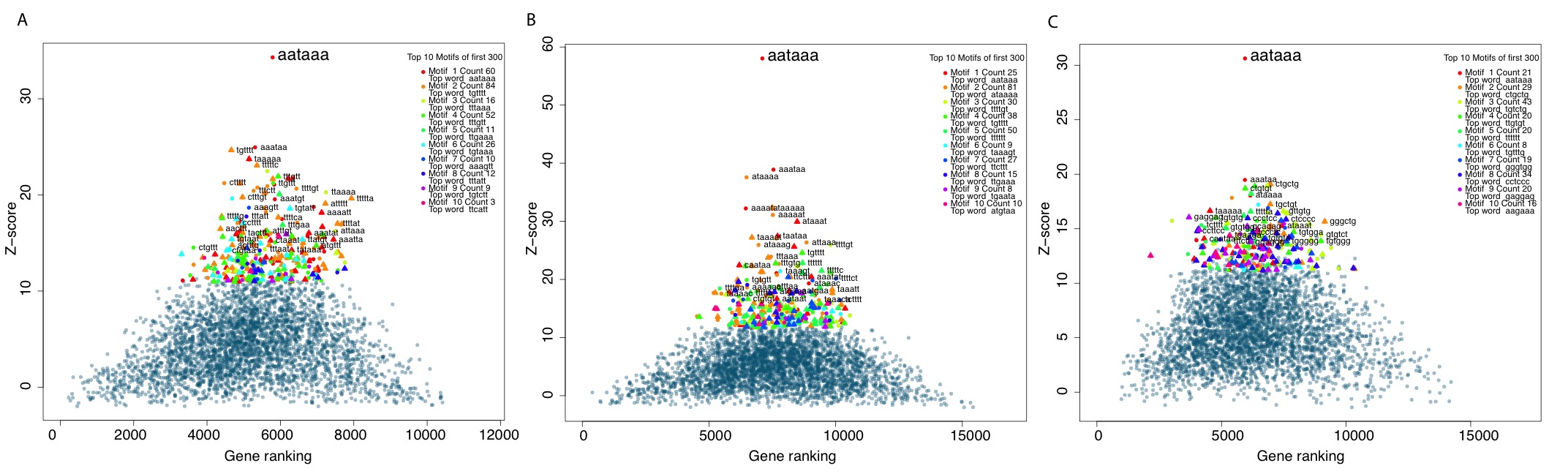


**Figure 4**: Motif analyses of WDR33 (A), CstF-64 (B) and Fip1 (C) reveal the strong PAS (AATAAA) enrichment in the sequences that overlap CLIP-cluster. The Most common weak PAS (ATTAAA) and TG-rich motifs also are significantly enriched in the CLIP clusters.

## References

1. Martin G, Gruber AR, Keller W, Zavolan M. Genome-wide analysis of pre-mRNA 3’ end processing reveals a decisive role of human cleavage factor I in the regulation of 3’ UTR length. Cell Rep. 2012;1: 753–63. doi:10.1016/j.celrep.2012.05.003

2. Schönemann L, Kühn U, Martin G, Schäfer P, Gruber AR, Keller W, et al. Reconstitution of CPSF active in polyadenylation: recognition of the polyadenylation signal by WDR33. Genes Dev. Cold Spring Harbor Laboratory Press; 2014;28: 2381–93. doi:10.1101/gad.250985.114

3. Rasmussen SH, Jacobsen A, Krogh A. cWords - systematic microRNA regulatory motif discovery from mRNA expression data. Silence. 2013;4: 2. doi:10.1186/1758-907X-4-2

4. R Developement Core Team. R: A Language and Environment for Statistical Computing. R Found Stat Comput. 2015;1: 409. doi:10.1007/978-3-540-74686-7
