# Supplementary figures and images for: Highly accessible AU-rich regions in 3’ untranslated regions are hotspots for binding of regulatory factors
Source: PLoS Comput Biol. 2017 Apr 14;13(4):e1005460. doi: 10.1371/journal.pcbi.1005460 (PMC5409497; doi:10.1371/journal.pcbi.1005460)

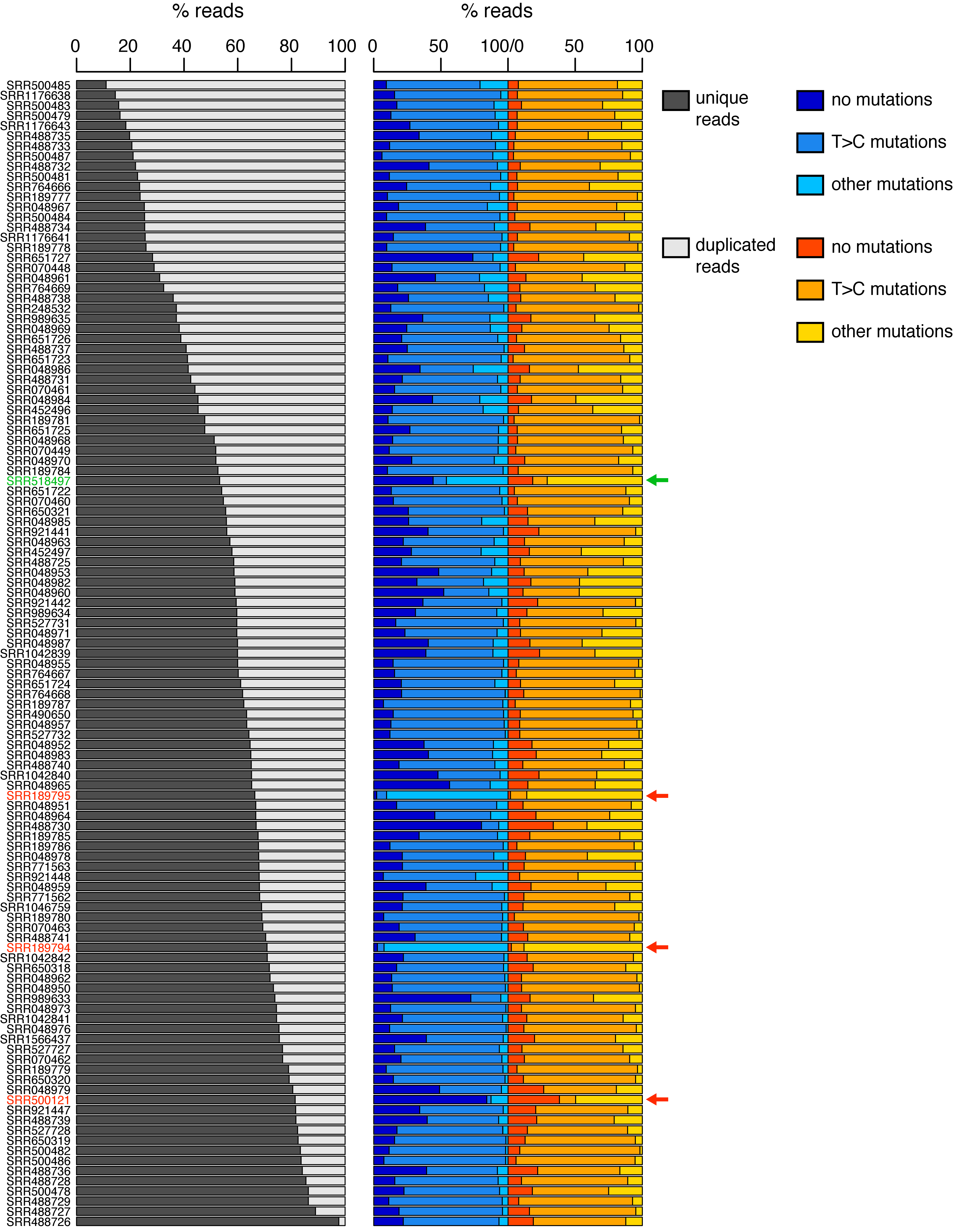

Supplement: S1 Fig — Mutation analysis of confidently mapped reads included in this study. For each library analyzed, we have classified reads according to whether there is only one read that maps to a particular genomic location (unique reads, dark grey) or multiple reads that map to the same location (duplicated reads, light grey). For each of these groups, we show in the right panel the % of reads that have no mutations, T>C conversions or other types of mutations. RNA-seq datasets and DGCR8 dataset, which are not expected to have high T>C mutation rates are highlighted in red and green respectively. (TIF) [file pcbi.1005460.s011.tif]

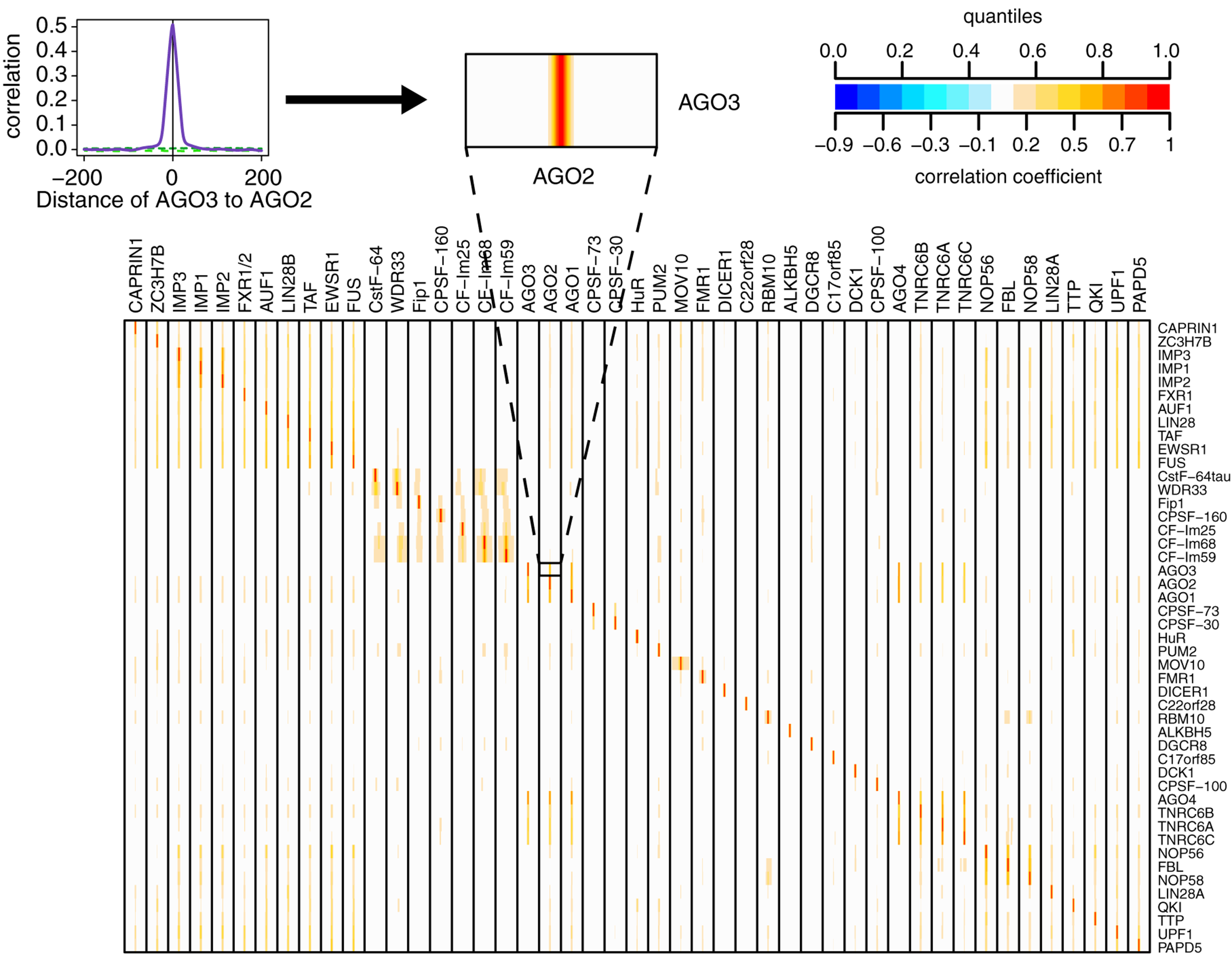

Supplement: S2 Fig — Pearson correlation coefficients of cluster enrichments are calculated for each pair of proteins (purple line) in a distance from -200 to 200 nt and represented using a color scale from blue (pearson correlation coefficient = -1) to red (pearson correlation coefficient = 1). All against all positional correlations calculated in this way are summarized in the heatmap (bottom). For each RBP in a column we show the pearson correlation coefficient of the RBPs in the rows around its binding sites. (TIF) [file pcbi.1005460.s012.tif]

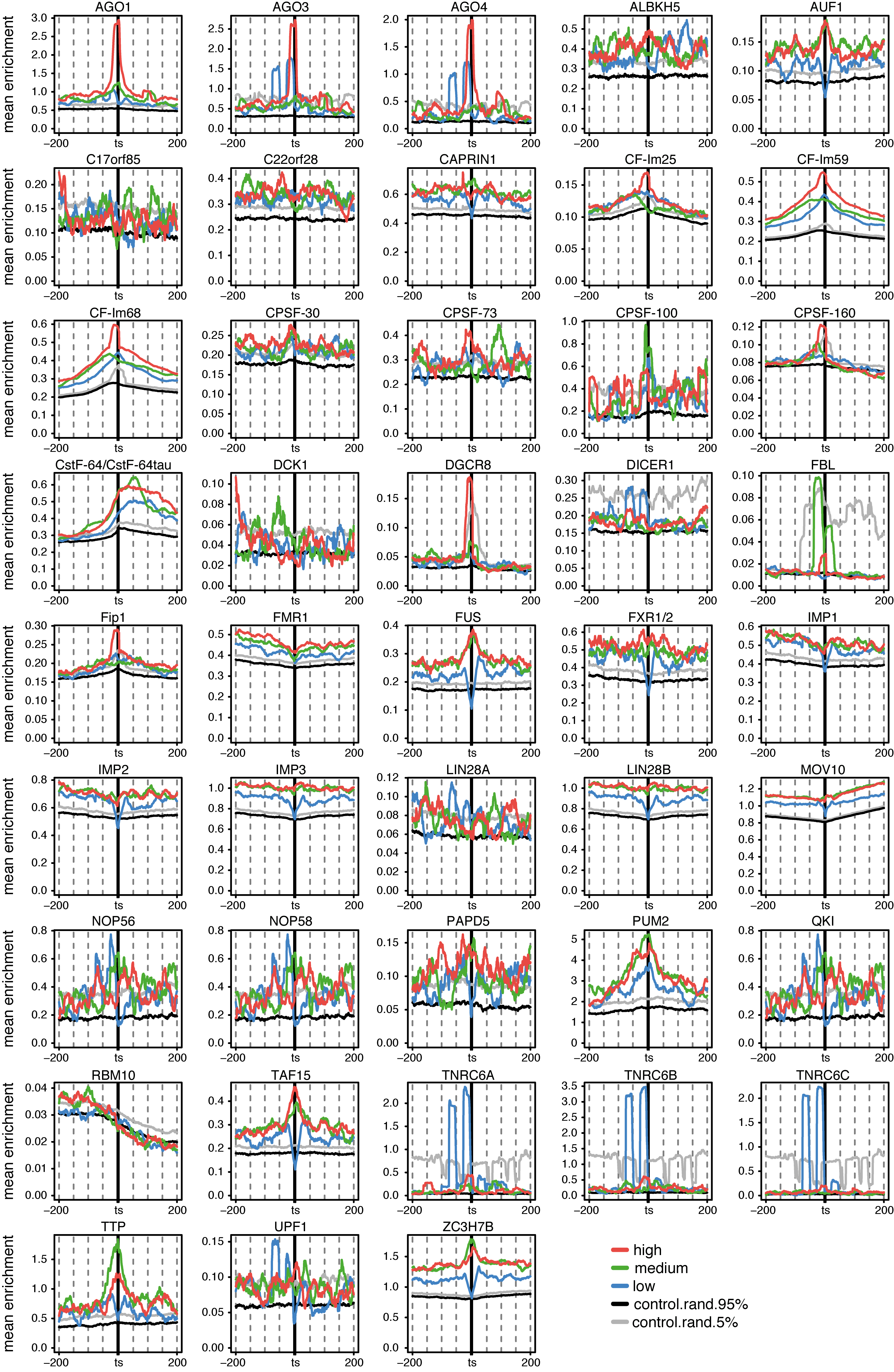

Supplement: S3 Fig — CLIP enrichment around miRNA target sites that are highly (red), moderately (green) and lowly (blue) expressed for the other 43 RBPs analyzed. The grey and the black lines show the maximum and the minimum enrichment values for the 90% confidence intervals around random target sites. (TIF) [file pcbi.1005460.s013.tif]

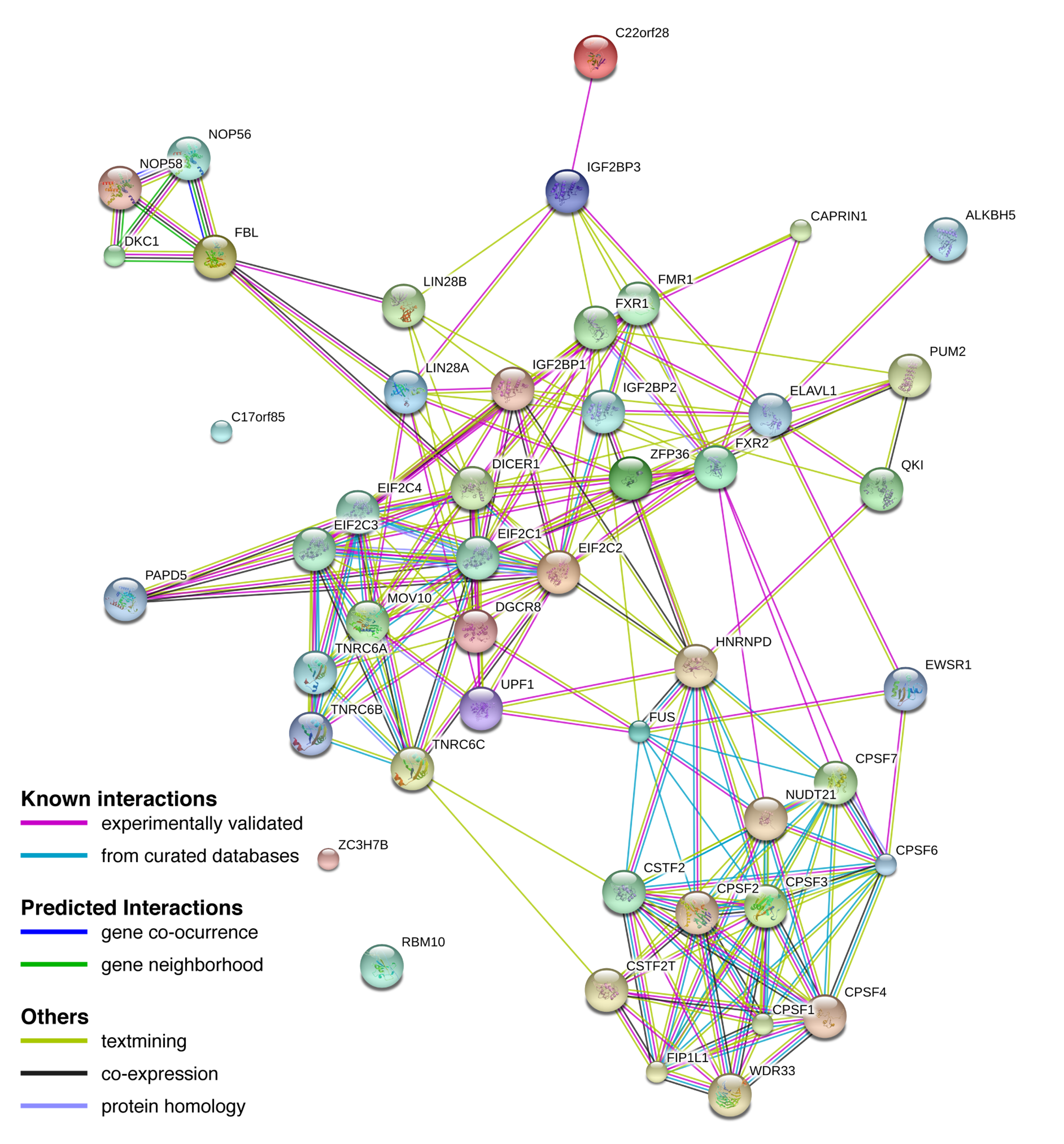

Supplement: S4 Fig — In this graph RBPs are depicted as nodes. Two RBPs are connected through edges if they interact according to STRING database. The color of the edges represent the type of interactions among them, which include known interactions, predicted interactions and other types of associations. (TIF) [file pcbi.1005460.s014.tif]

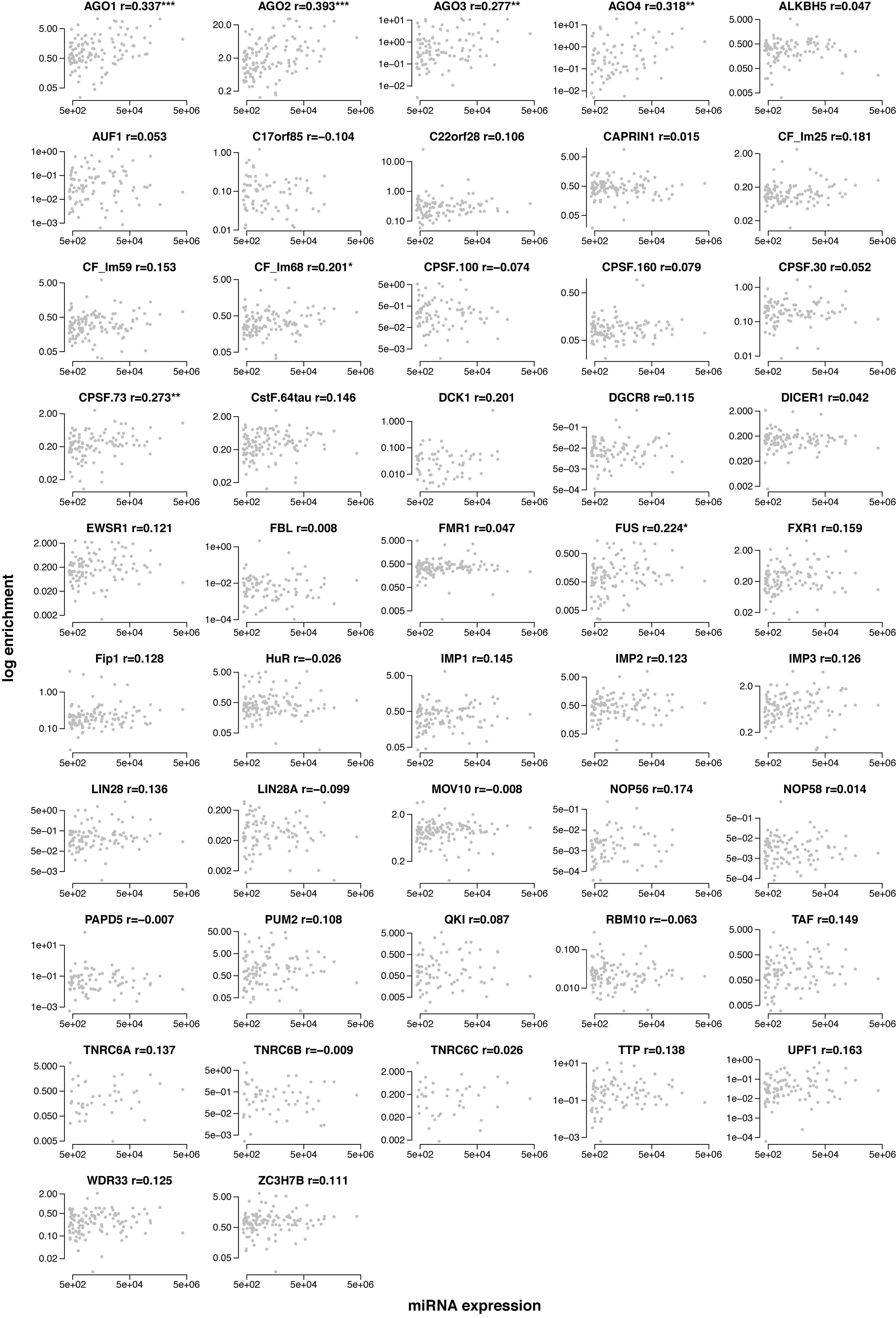

Supplement: S5 Fig — Scatter plots showing the relation between CLIP enrichment at miRNA target sites (y-axis) and the expression of miRNAs targeting them (x-axis). Axes are shown in log scale. For each RBP, the Pearson correlation coefficient r is shown. Significant correlations are marked as *, ** or *** corresponding to p-values < 0.05, < 0.01 and < 0.001 respectively. (TIF) [file pcbi.1005460.s015.tif]

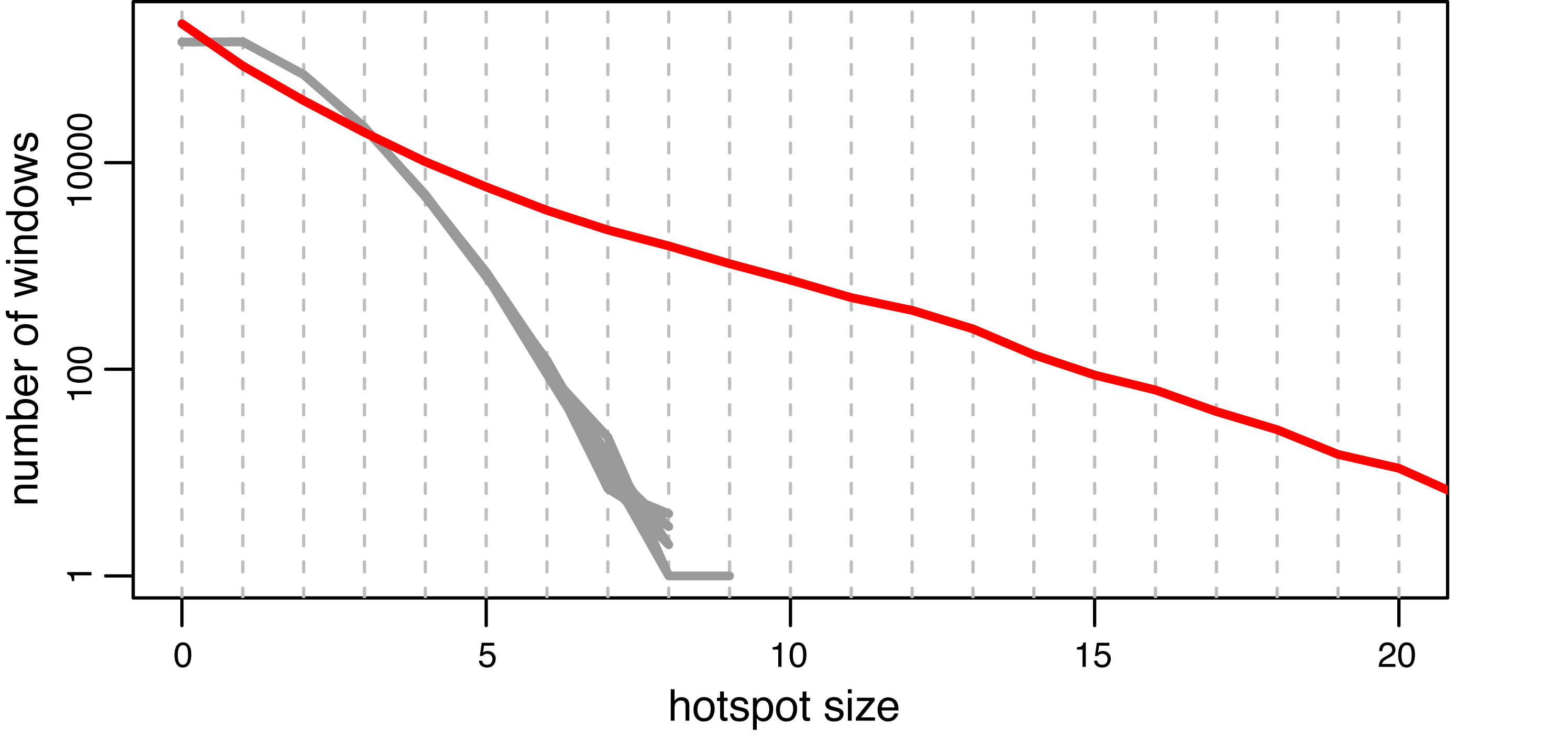

Supplement: S6 Fig — Hotspot size distribution in simulated data (grey lines) and real data (red line). The x-axis shows the hotspot size and the y-axis the count of windows containing hotspots with a specific amount of RBPs. (TIF) [file pcbi.1005460.s016.tif]

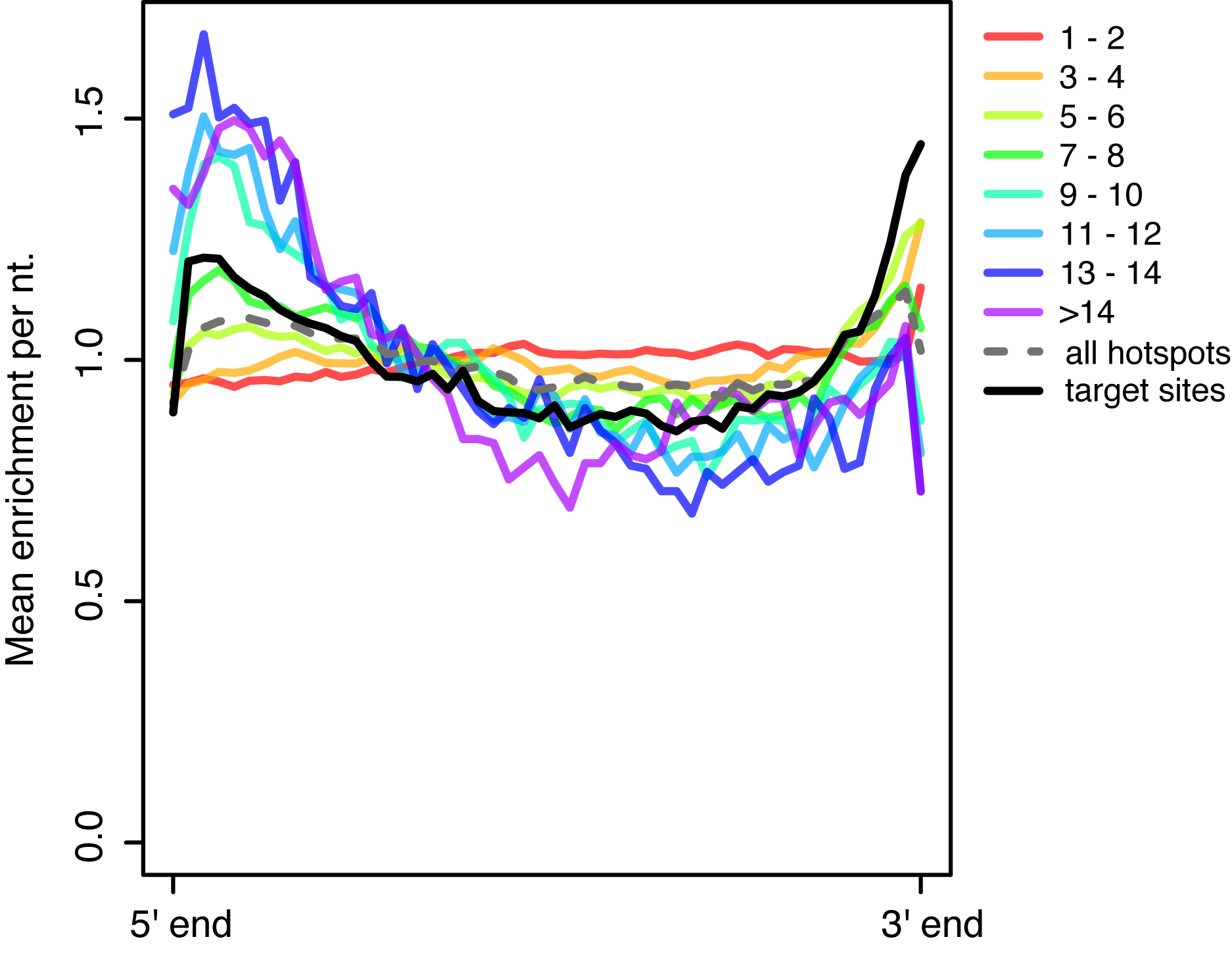

Supplement: S7 Fig — Each colored line shows the ratio of the amount of windows with a specific amount of RBPs in a bin relative to the average amount of windows with that specific amount of RBPs across bins. The distribution of all hotspots, i.e. windows with more than 4 RBPs, is shown with a dashed grey line. For each line, the y-axis shows the enrichment relative to the mean of the line whereas the x-axis shows the position of the bin across a length-normalized 3’UTR. For comparison, the enrichment of miRNA target sites relative to the mean number of target sites per bin is shown. (TIF) [file pcbi.1005460.s017.tif]

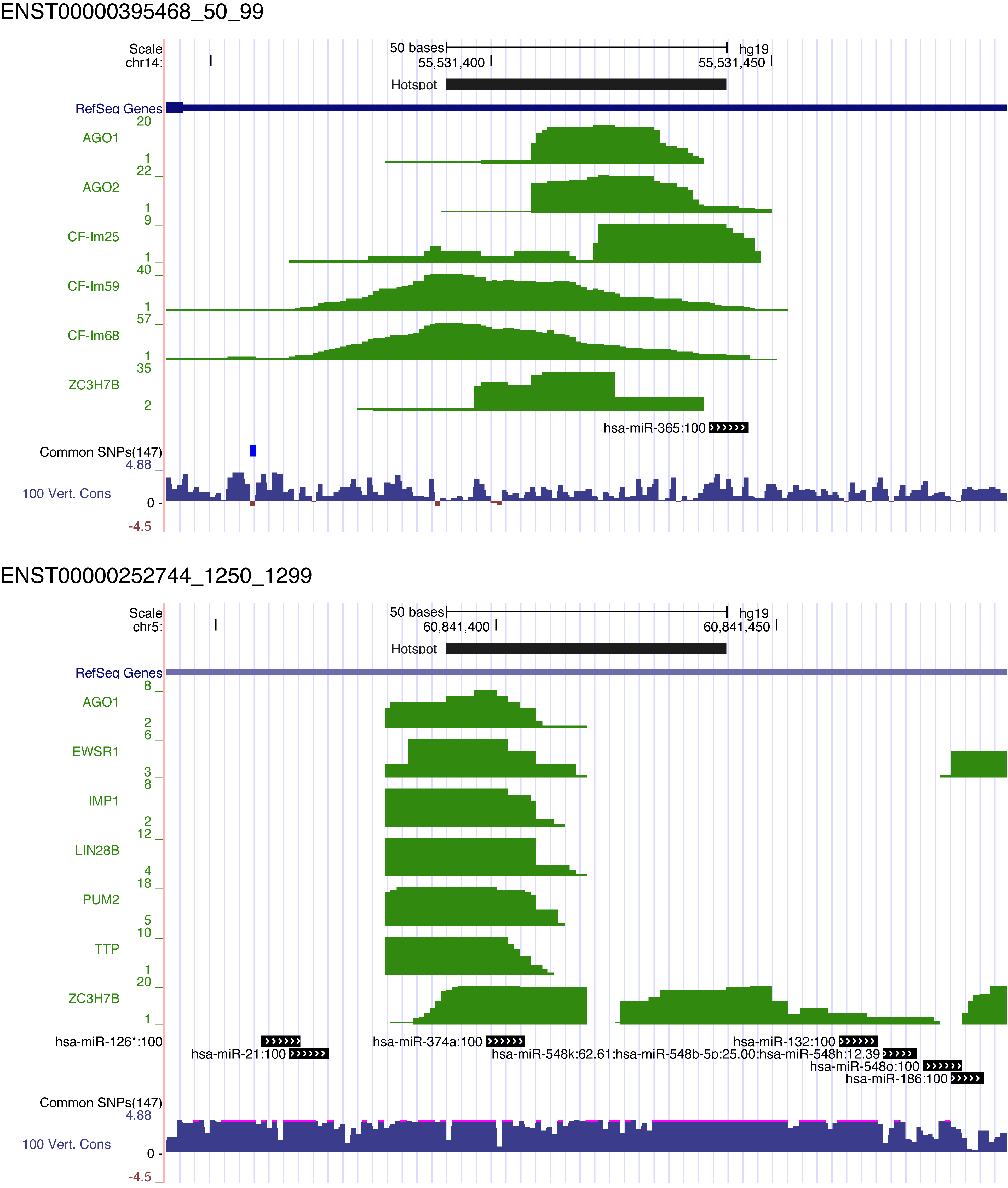

Supplement: S8 Fig — UCSC screenshots displaying the binding of several RBPs on two of the hotspots identified. For each of the RBP tracks, the height of the tracks represents the amount of CLIP reads binding in a particular location. Additionally, SNPs, miRNA target sites used in this paper and phyloP scores are shown. (TIF) [file pcbi.1005460.s018.tif]

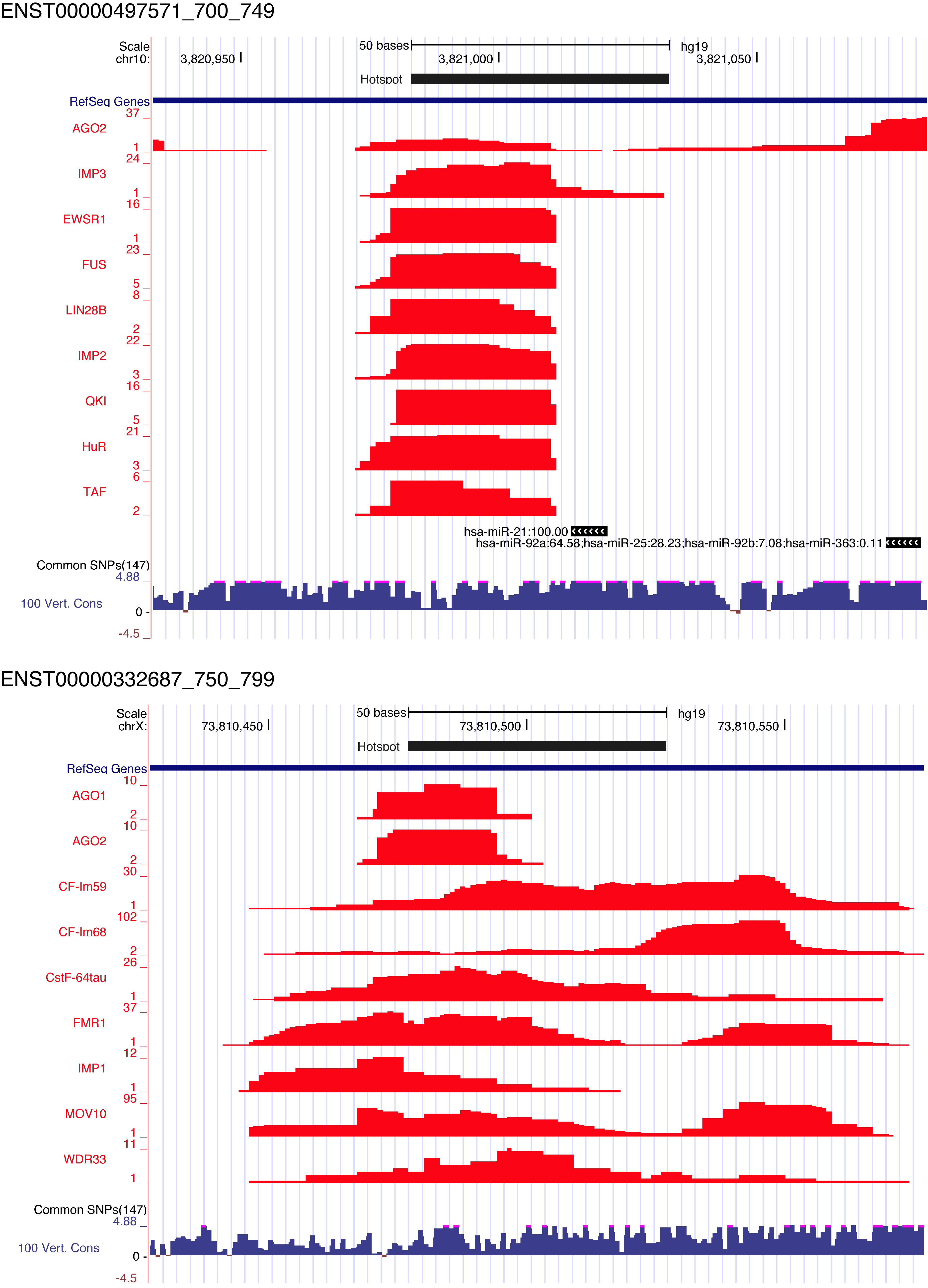

Supplement: S9 Fig — UCSC screenshots displaying the binding of several RBPs on two of the hotspots identified. For each of the RBP tracks, the height of the tracks represents the amount of CLIP reads binding in a particular location. Additionally, SNPs, miRNA target sites used in this paper and phyloP scores are shown. (TIF) [file pcbi.1005460.s019.tif]

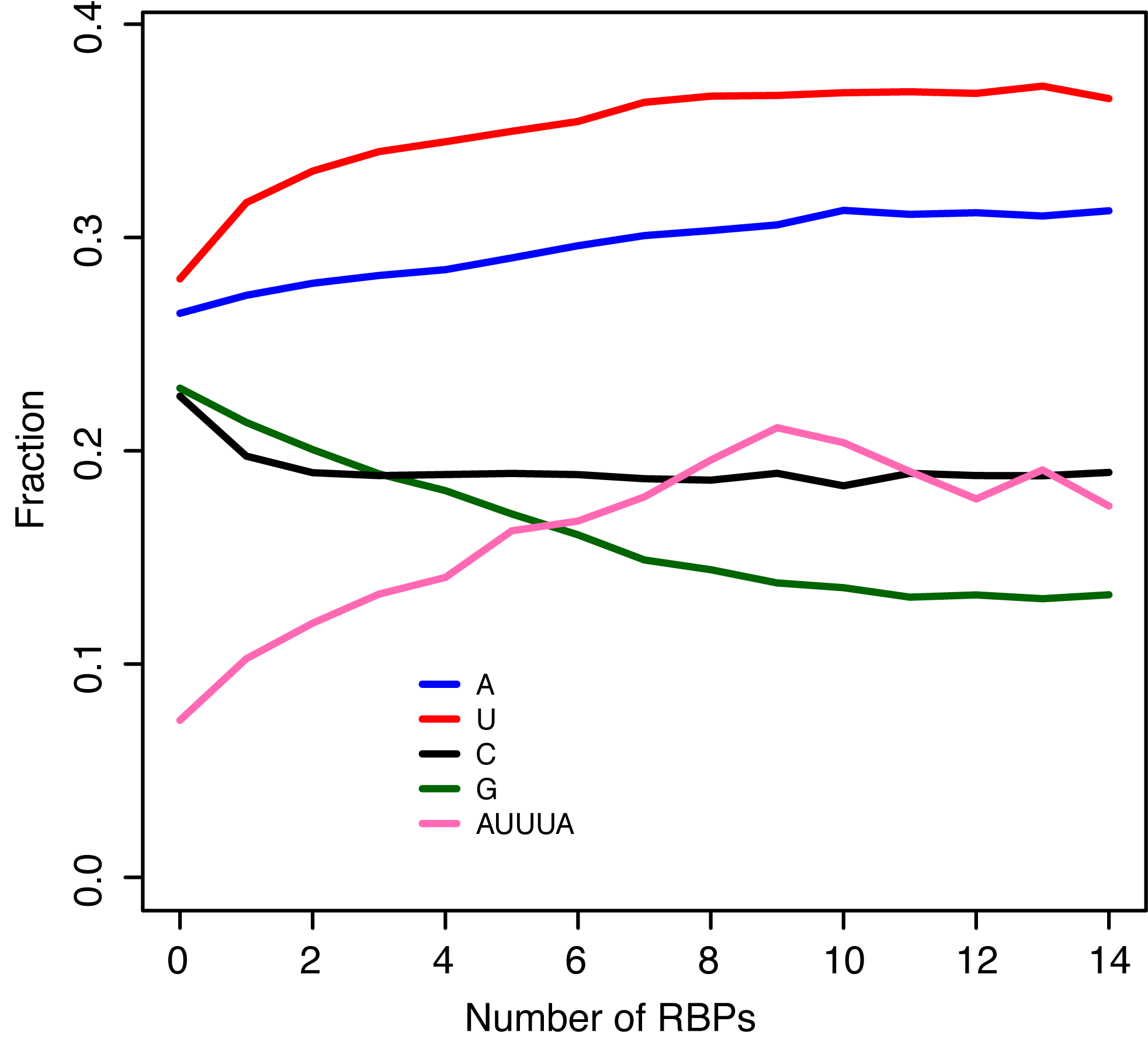

Supplement: S10 Fig — The fraction of A, U, C and G nucleotides in a window is represented by a red, blue, black and pink line respectively. The x-axis shows the number of RBPs in a window. The y-axis shows the fraction of nucleotides in the window. Additionally, the green line shows the fraction of the windows that contain 1 or more occurrences of the core ARE AUUUA. (TIF) [file pcbi.1005460.s020.tif]

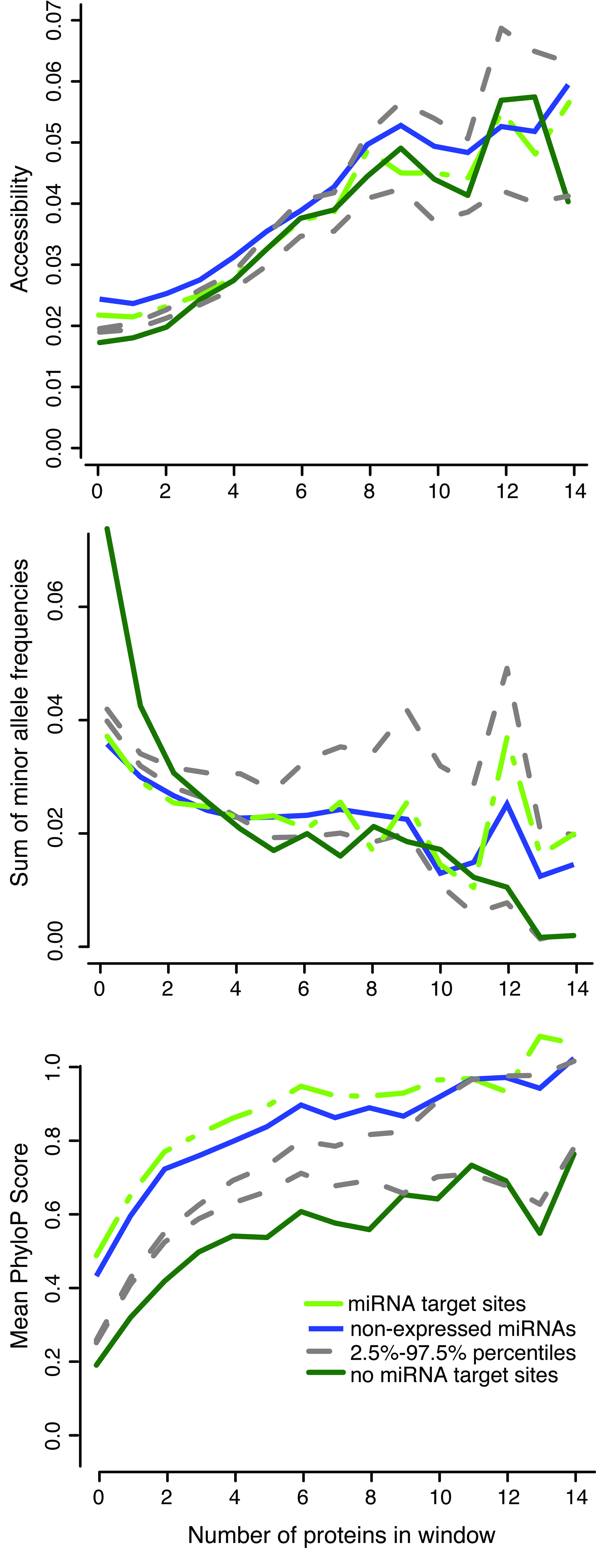

Supplement: S11 Fig — Relation between accessibility (top), sum of minor allele frequencies (middle), and conservation (bottom) with the number of RBPs in a window after excluding all the windows that overlap background GFP clusters. The lines show the behavior of windows that overlap expressed miRNAs in HEK293 (light green), non-expressed miRNAs in HEK293 (blue) or that do not overlap miRNA target sites (dark green). The grey dashed lines mark the 2.5% and 97.5% percentiles of the distribution obtained by generating 100 random miRNA datasets. The presence of miRNA target sites in the windows does not affect the correlation observed between accessibility and sum of minor allele frequencies. In contrast, windows that overlap miRNA target sites (both expressed and non-expressed) show higher conservation regardless of the amount of RBPs that bind in the window. All the features analyzed show significant correlations of similar magnitude to those observed in the whole dataset. (TIF) [file pcbi.1005460.s021.tif]

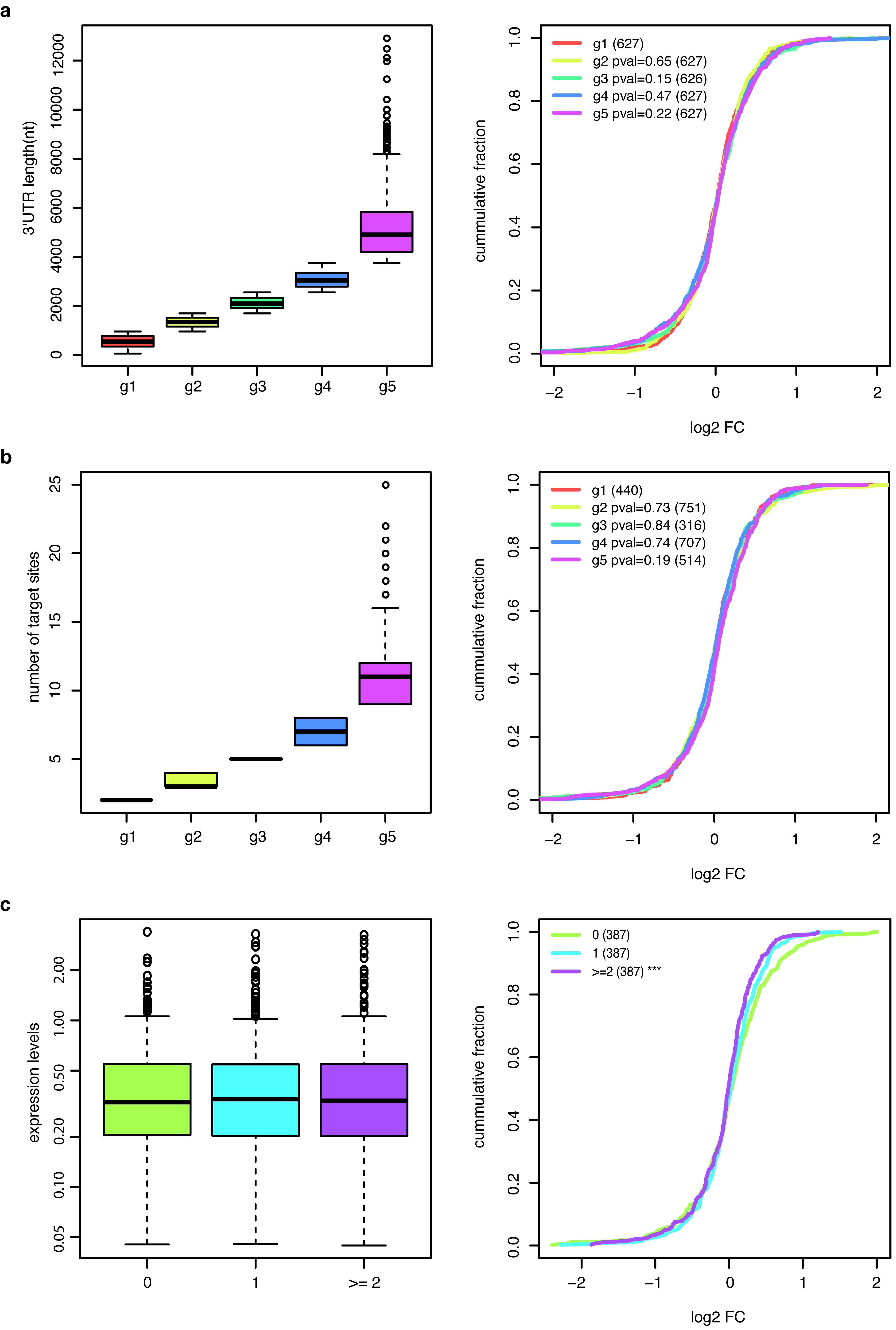

Supplement: S12 Fig — Correlation analysis between log2 fold changes and a) 3’UTR length and b) number of target sites in the 3’UTR. Transcripts were divided in 5 equally sized groups (left plot) according to their 3’UTR length or the number of target sites and for each of the groups the cumulative distribution functions of the log2 fold changes are displayed (right plot). No significant differences are found between contiguous groups of transcripts (KS test). c) Comparison of log2 fold changes across expression matched gene sets (left) with 0, 1 or 2 or more hisites overlapping RBP hotspots (right). In the left panel, the boxplots summarize the average expression of the genes in each of the groups measured using microarray data. For each of the original datasets, 387 genes were sampled so that the expression distribution was preserved. Upon AGO2 KD, significant differences (KS test p-value < 0.01) are only observed for the set with 2 or more hisites covered by hotspots compared to the set with free hisites. (TIF) [file pcbi.1005460.s022.tif]

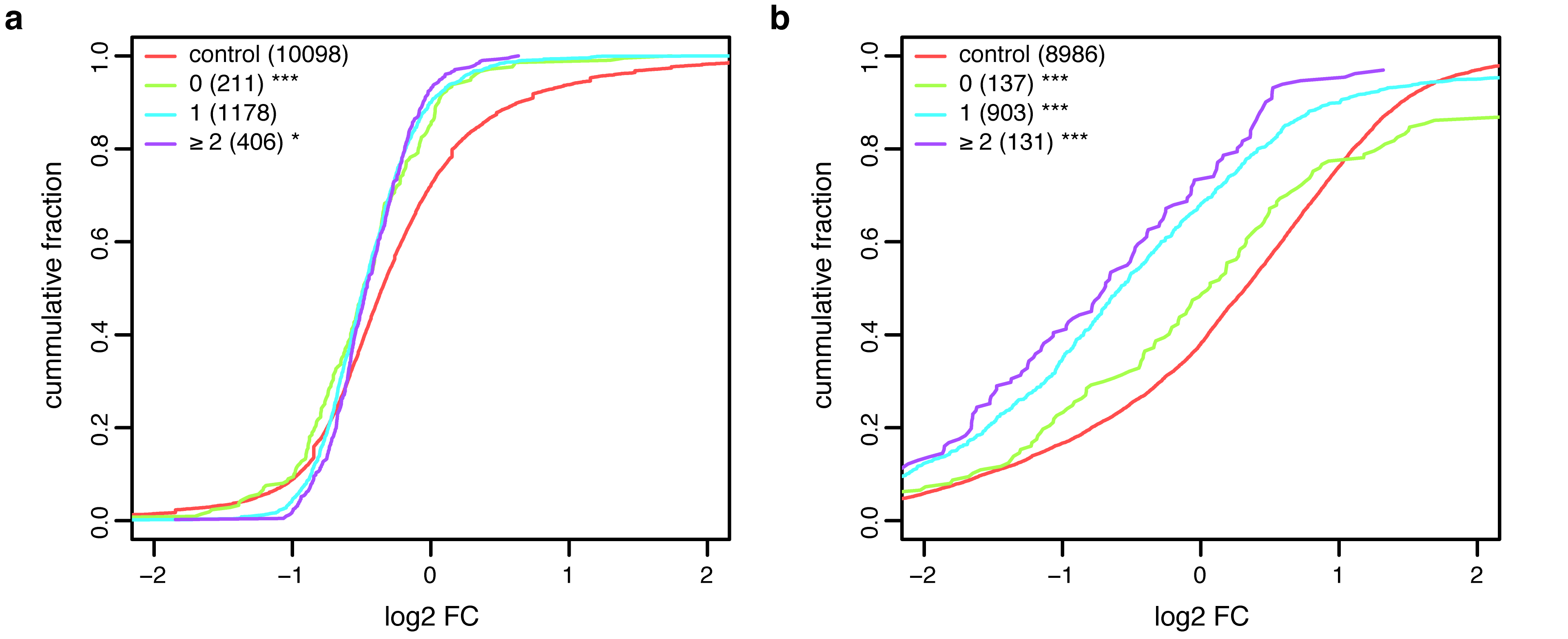

Supplement: S13 Fig — Cumulative fraction plot showing the effect of having 0, 1 or 2 or more a) TTP binding sites or b) AUF binding sites overlapping RBP hotspots. The x-axis shows the distribution of log2FC upon KD of AUF or TTP respectively, and the y-axis shows the cumulative fraction of genes. As a control, genes with a) no TTP and b) no AUF binding sites are shown. *, ** and *** denote a p-value < 0.05, 0.01 and 0.001 respectively. The sets containing 1 or more sites overlapping hotspots are compared to set without sites overlapping hotspots. The set without sites overlapping hotspots is compared to the control set. (TIF) [file pcbi.1005460.s023.tif]

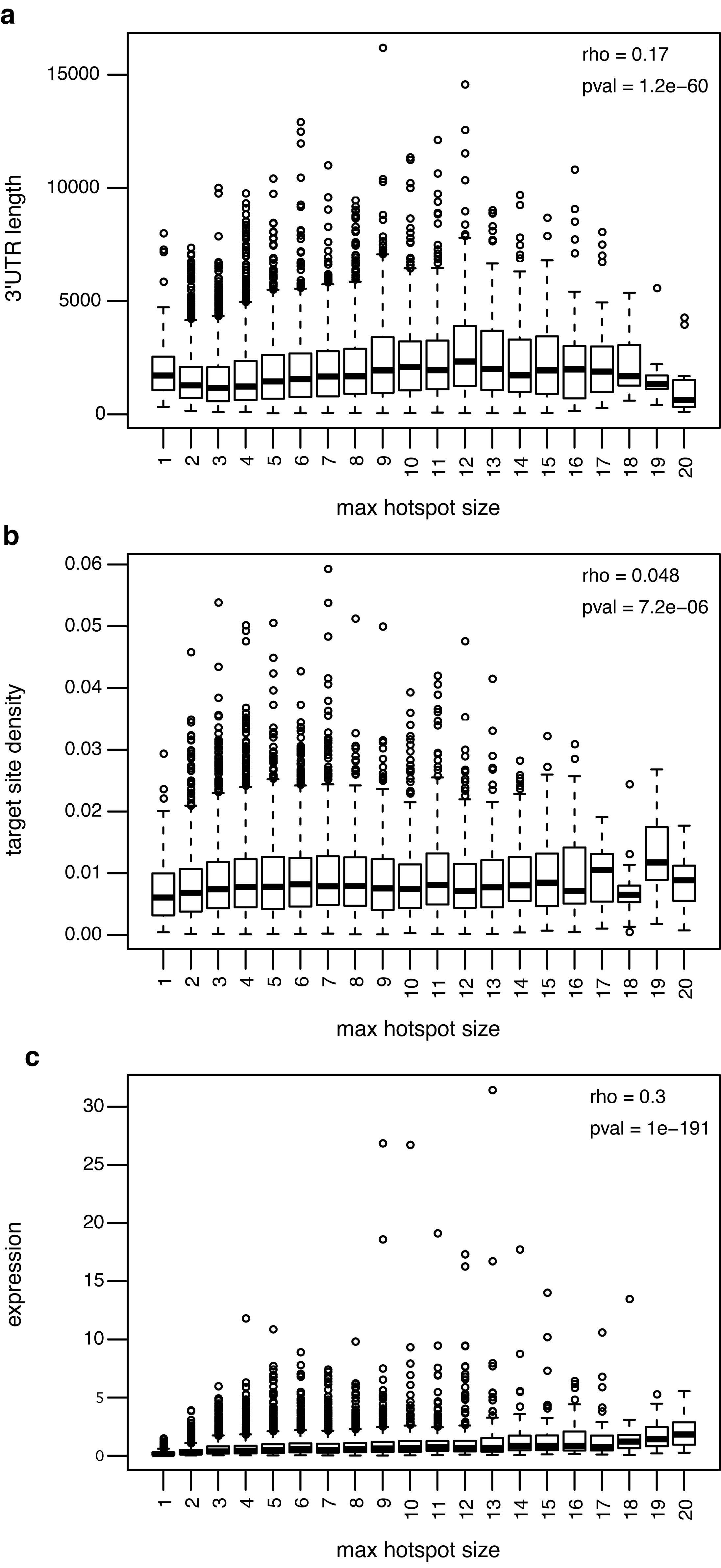

Supplement: S14 Fig — Boxplot distribution showing the correlation between the maximum hotspot size in a transcript (x-axis) and a) 3’UTR length, b) target site density and c) expression levels. The spearman correlation coefficient rho and the p-value of the correlation are shown. (TIF) [file pcbi.1005460.s024.tif]

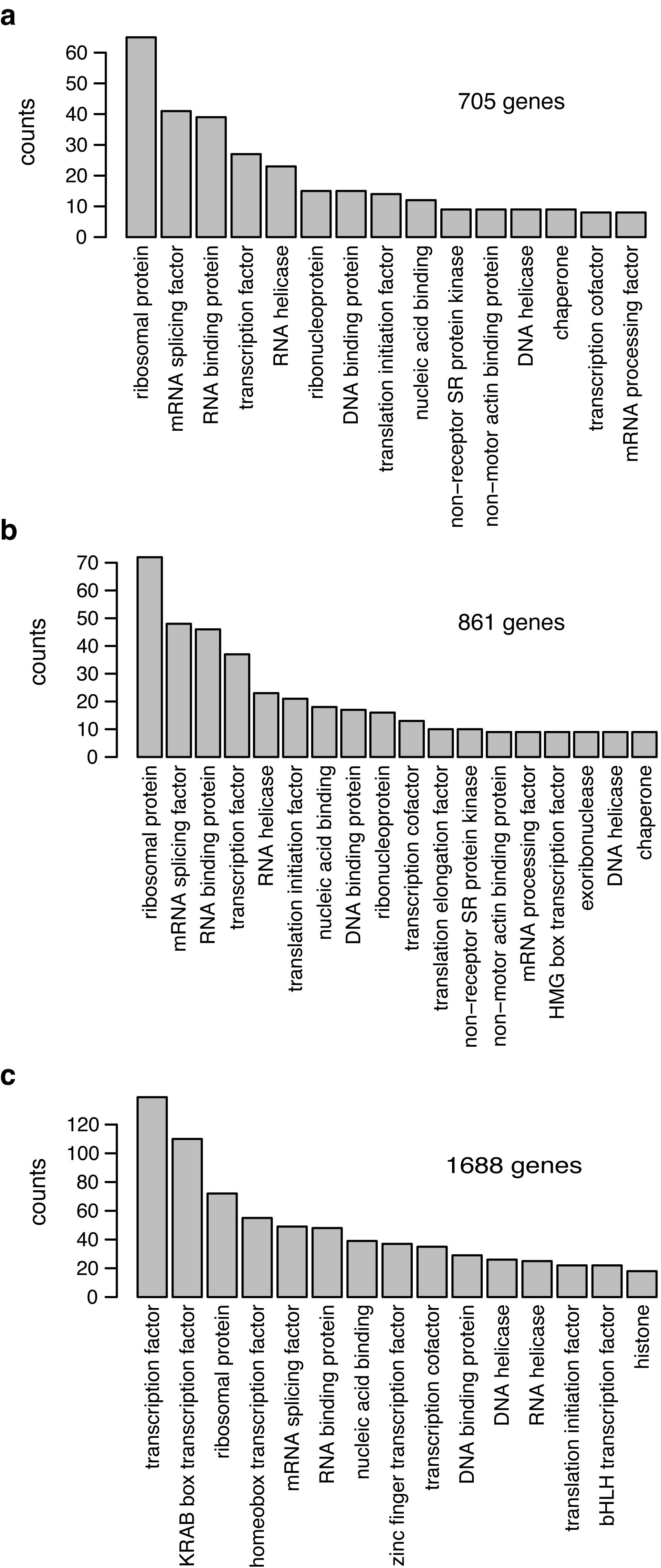

Supplement: S15 Fig — Barplot distribution of the most abundant PANTHER protein classes in genes containing hotspots annotated with the significant GO terms a) PolyA RNA binding b) RNA binding and c) nucleic acid binding. For each of the plots, the number of genes in each of the groups is given. Only those protein classes present in at least 1% of the genes in each category are shown. (TIF) [file pcbi.1005460.s025.tif]

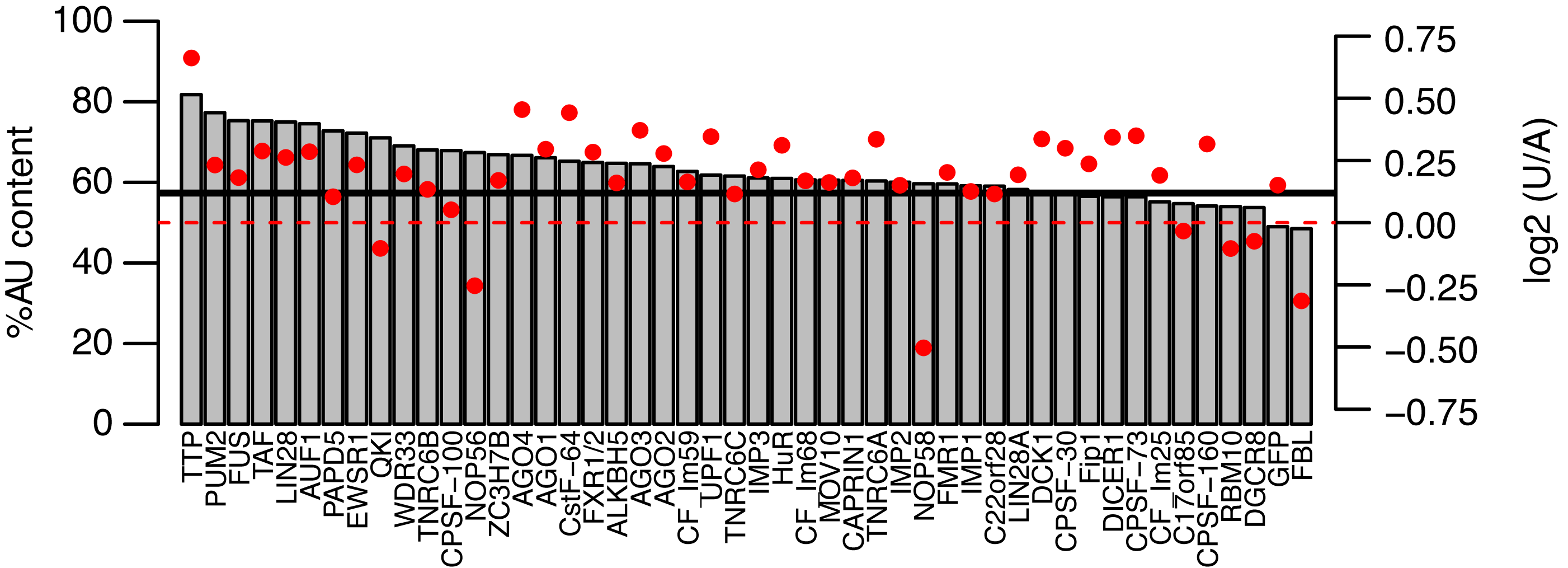

Supplement: S16 Fig — Barplot showing the % of AU content of the significant clusters identified in 3’UTRs for all the CLIP datasets analyzed compared to general AU-content of 3’UTRs (black line; left axis). Red dots indicate log2 ratio of U vs A content for each RBP, which is higher than the bias observed in whole 3’UTRs (dashed red line; right axis). Most datasets show a higher AU bias compared to general 3’UTRs, which is mainly due to an increase proportion of Us in the sequence, probably due to a technical bias introduced by PAR-CLIP. (TIF) [file pcbi.1005460.s026.tif]

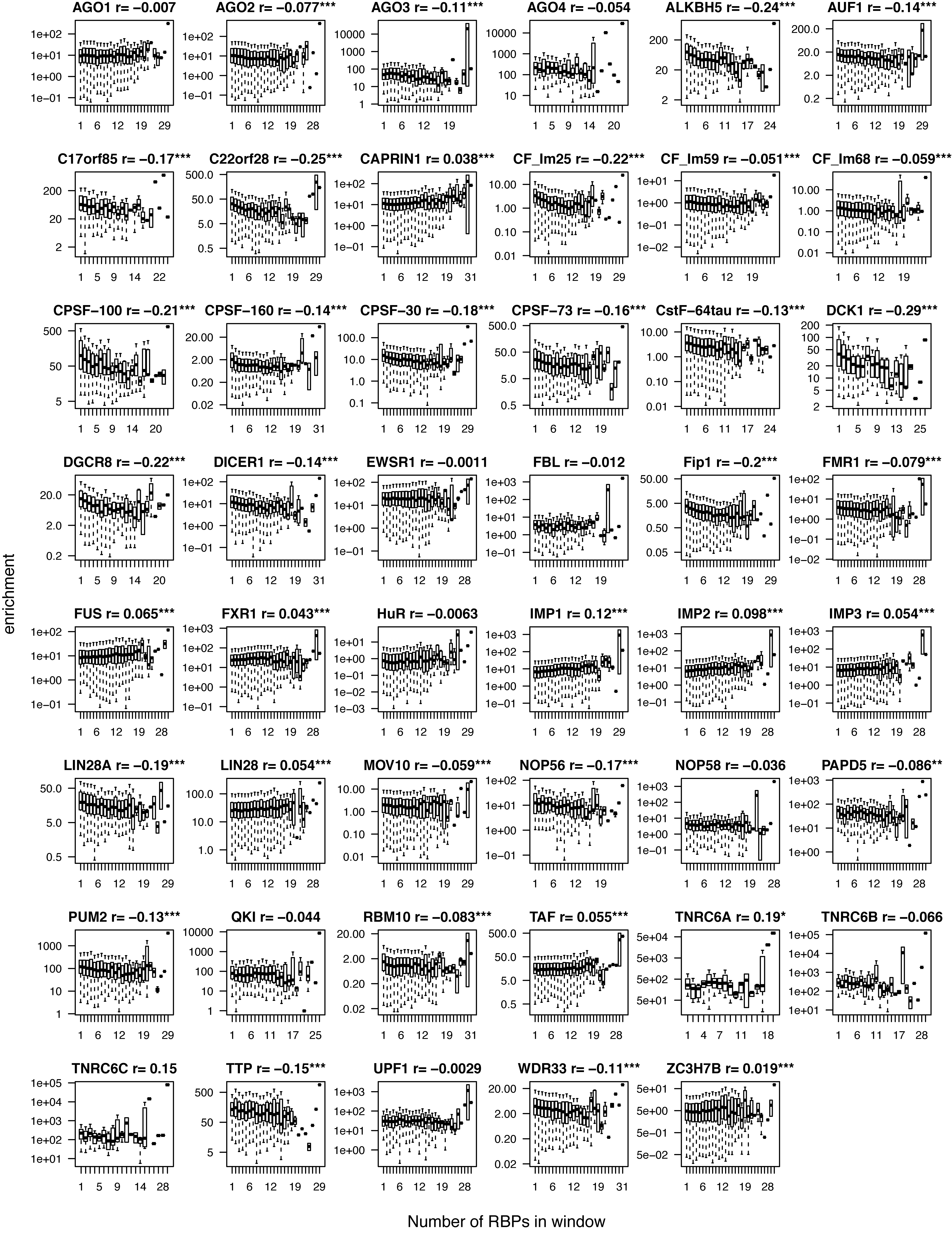

Supplement: S17 Fig — Boxplots showing the relation between the number of RBPs in a hotspot (x-axis) and RBP enrichment on hotspots (y-axis) for all RBPs analyzed. For each RBP, the Pearson correlation coefficient r is shown. Significant correlations are marked as *, ** or *** corresponding to p-values < 0.05, 0.01 and 0.001. (TIF) [file pcbi.1005460.s027.tif]
